# Future applications of fluorescence lifetime imaging ophthalmoscopy in neuro-ophthalmology, neurology, and neurodegenerative conditions

**DOI:** 10.3389/fneur.2025.1493876

**Published:** 2025-03-07

**Authors:** Daniel M. Markowitz, Elizabeth Affel, György Hajnóczky, Robert C. Sergott

**Affiliations:** ^1^Drexel University College of Medicine, Philadelphia, PA, United States; ^2^William H. Annesley, EyeBrain Center, Vicky and Jack Farber Neuroscience Institute, Thomas Jefferson University, Partnered with Wills Eye Hospital, Philadelphia, PA, United States; ^3^MitoCare Center, Department of Pathology, Anatomy and Cell Biology, Thomas Jefferson University, Philadelphia, PA, United States

**Keywords:** neurodegenerative diseases, fluorescence lifetime imaging ophthalmoscopy, Alzheimer’s disease, Parkinson’s disease, neuromyelitis optica spectrum disorder

## Abstract

Fluorescence lifetime imaging ophthalmoscopy (FLIO) has emerged as an innovative advancement in retinal imaging, with the potential to provide *in vivo* non-invasive insights into the mitochondrial metabolism of the retina. Traditional retinal imaging, such as optical coherence tomography (OCT) and fundus autofluorescence (FAF) intensity imaging, focus solely on structural changes to the retina. In contrast, FLIO provides data that may reflect retinal fluorophore activity, some of which may indicate mitochondrial metabolism. This review builds upon the existing literature to describe the principles of FLIO and established uses in retinal diseases while introducing the potential for FLIO in neurodegenerative conditions.

## 1 Introduction

Although fundus photography and intravenous fluorescein angiography were originally developed for retinal diseases, these imaging techniques have provided valuable insights into the diagnosis, treatment, and pathogenesis of neurological and neuro-ophthalmic conditions (1–9). These observations have validated the hypothesis that the retina is as much a part of the central nervous system as the optic nerves, chiasm, and structures merging visual information with memory, depth perception, and other higher cortical functions.

Spectral-domain optical coherence tomography (OCT) and optical coherence tomography angiography (OCT-A), the most widely used retinal imaging technologies in both clinical practice and clinical trials, have revealed previously hidden details of the structure of retinal layers as well as the retinal microvascular circulation (10–14). For some years, fundus autofluorescence (FAF) intensity imaging was used to evaluate the metabolic status of the retina indirectly. However, fundus autofluorescence intensity measurements are prone to media opacities, high image noise, low contrast, and do not allow the assignment of the signal to specific chemical compounds. Furthermore, compounds with bright autofluorescence, such as lipofuscin, might overwhelm the fluorescence signal of other compounds (15). Thus, these methods have significant limitations in evaluating the *in vivo* metabolism of the healthy retina or the retina afflicted with primary ophthalmic or neurological diseases.

Approximately 10 years ago, peer-reviewed reports described and defined fluorescence lifetime imaging ophthalmoscopy (FLIO), the clinical equivalent of fluorescence lifetime imaging microscopy (FLIM). FLIO offers non-invasive, non-contact, and reproducible insights into the metabolic activity of the retina through fluorophores such as flavin adenine dinucleotide (FAD), lipofuscin, lutein, zeaxanthin, and meso-zeaxanthin. However, further validation is required for its broader clinical applications (15–17). Just as positron emission tomography (PET) scanning adds metabolic dimensions to MRI, we believe that FLIO offers the *in vivo* physiological counterpart to OCT.

**Figure 1:**
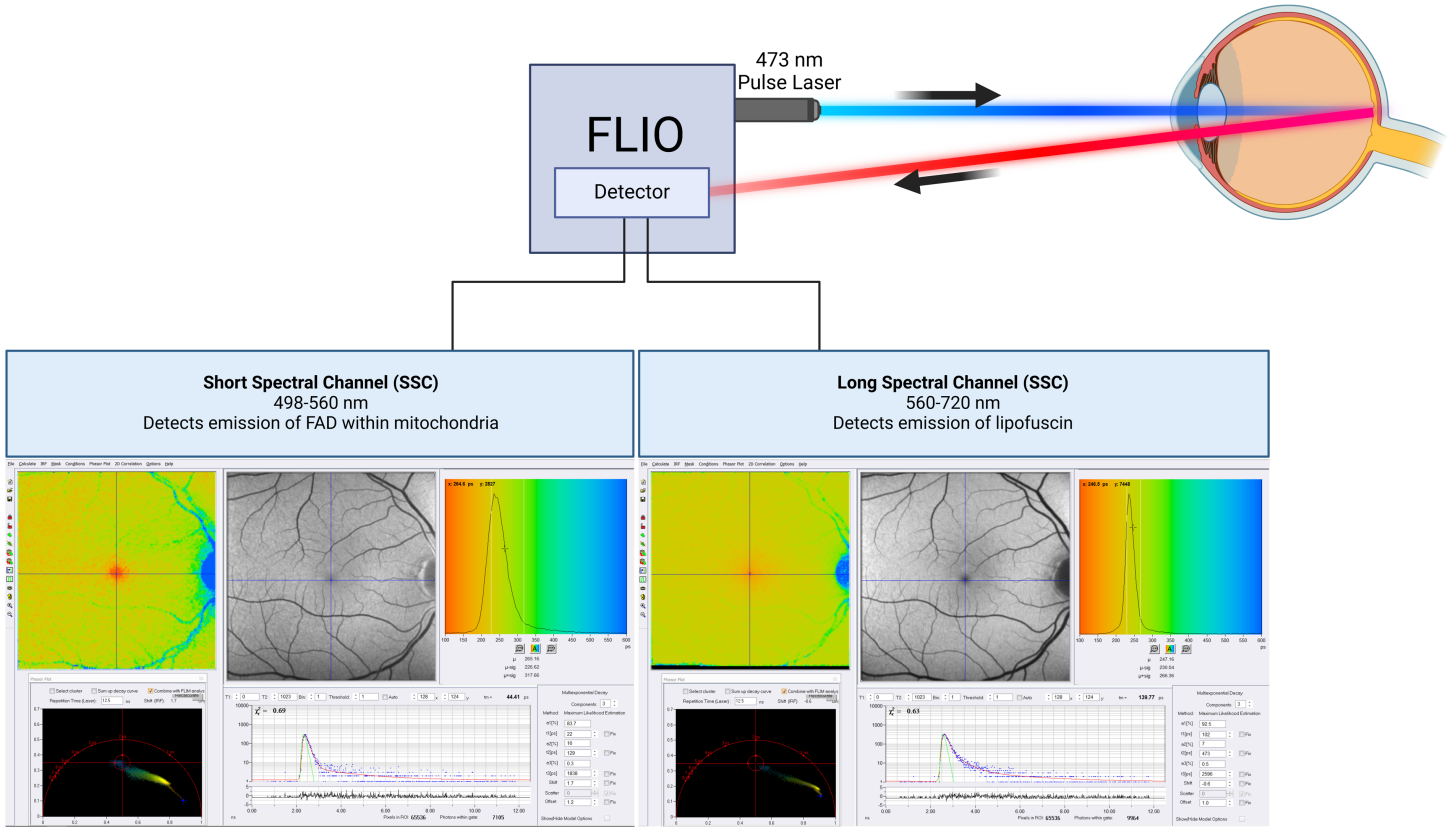
Schematic of FLIO imaging. The technical description of FLIO equipment in this review is on the Heidelberg Spectralis based FLIO instrument. The machine uses two hybrid photon-counting detectors (HPM-100-40, Becker&Hickl GmbH, Berlin, Germany) to record fluorescence photons from each pixel across 1,024 time channels. This design generates two separate photon arrival histograms for each pixel, one for each spectral channel. The photon arrival histograms are then used to calculate fluorescence lifetimes. This equipment was used in almost all cited studies. This figure was created with BioRender.com.

FLIO measures the fluorescence decay over time of metabolically active tissues without fluorescent dyes (16, 18) (Figure 1). Different molecules within each subcellular structure display unique auto-fluorescent signatures depending upon the molecular environment. However, the measured lifetime may represent contributions from multiple fluorophores, and the attribution of fluorescence lifetimes to specific molecules requires further investigation. FLIO can potentially provide valuable insights into mitochondrial oxidative metabolism and lysosomal function. However, isolating specific retinal fluorophores from these organelles *in vivo* remains challenging due to overlapping emission spectra. Advanced *in vitro* techniques such as FLIM may aid in validating and isolating specific signals (17, 19). Although standard FLIO currently does not allow for the differentiation of signal sources based on retinal layer dependence, future integration with spectral domain OCT may enable the identification of layer-specific fluorescence lifetime signals, providing greater insight into the localization of fluorophores within the retina. Using FLIO and spectral domain OCT, we expect to fulfill the critical paradigm of merging function with structure.

In the early 2000s, FLIO was first investigated by Dietrich Schweitzer and Martin Hammer using a prototype device to image the human retina (20–25). The technology Schweitzer and Hammer developed was advanced by Heidelberg Engineering in Germany and Switzerland in 2012 (16). In dermatology, fluorescence lifetime imaging has already enabled the detection of metabolic changes within basal cell carcinomas and surrounding tissue compared to healthy surrounding tissue (26). In cardiology, fluorescence lifetime imaging has been used to define the heterogeneity of atherosclerotic plaques and identify plaque disruption in coronary heart disease (16, 27–29).

## 2 Principles of FLIO

### 2.1 Basic principles of fluorescence

Fluorescence is defined as the emission of visible or invisible electromagnetic radiation by a substance after exposure to external radiation of a shorter wavelength, such as X-rays or ultraviolet light. In other words, fluorophore, a chemical compound or molecule, absorbs light of short wavelengths and emits light of longer wavelengths. When a fluorophore absorbs a photon of one wavelength, it raises the molecule to an excited state for a short period before losing the energy as the emission of a photon, creating fluorescence as the molecule returns to its ground state. An individual “fluorophore lifetime” is the average time a molecule remains excited before emitting a photon and returning to its ground state. Fluorescence can be characterized by its intensity and lifetime. The fluorescence intensity signal is sensitive to several factors, including photobleaching, which do not affect the lifetime measurements.

Fluorescence lifetimes are depicted graphically in a log-linear fashion where shorter lifetimes exhibit a faster decay with a steeper slope, and longer lifetimes exhibit a more gradual slope. Various endogenous molecules contain specific fluorophores that emit fluorescence when excited. The fluorescence lifetime can be measured for individual fluorophores in isolation *in vitro*. In retinal tissues and the eye, autofluorescence is composed of many fluorophores, each with a different fluorescence lifetime, producing multiexponential decays. A simple measure of the multiexponential decay is the mean fluorescence lifetime, τ_m_.

### 2.2 FLIM and fluorescence

**Figure 2:**
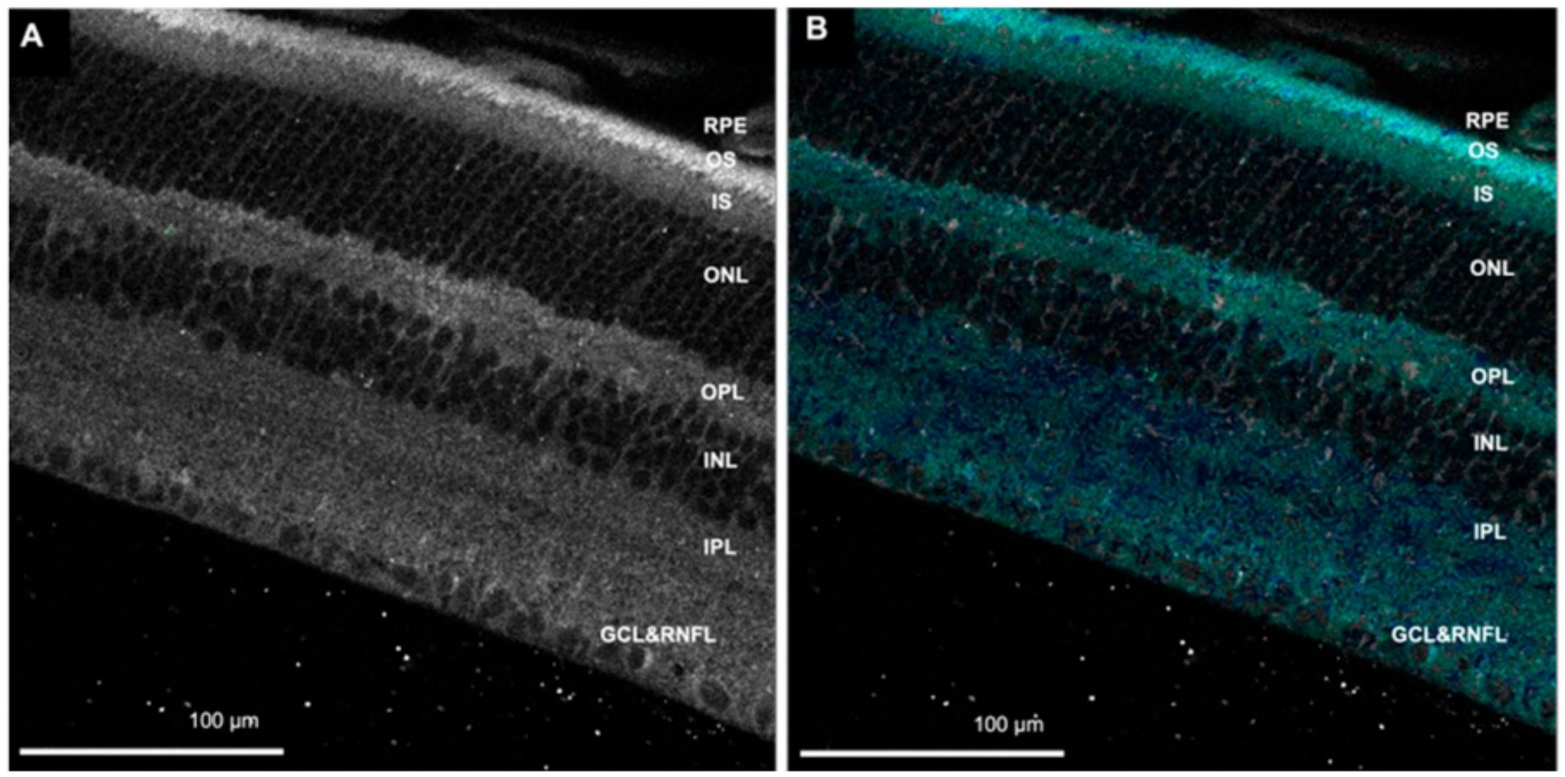
**(A)** Autofluorescence image of the retina. **(B)** Fluorescence lifetime image showing cyan/blue color corresponds with flavin adenine dinucleotide (FAD). RNFL, retinal nerve fiber layer; GCL, ganglion cell layer; IPL, inner plexiform layer; INL, inner nuclear layer; OPL, outer plexiform layer; ONL, outer nuclear layer; IS, photoreceptor inner segment; OS, photoreceptor outer segment; RPE, retinal pigment epithelium. Adapted from Kesavamoorthy et al. (38).

FLIO has evolved from FLIM, which evaluates biological mechanisms at the molecular level in various tissues or cells on the microscope stage (19). In FLIM studies, both autofluorescence lifetime and the lifetime of fluorescent dyes/fluorescent proteins introduced to the sample to monitor specific parameters such as glucose, ATP, or Ca^2+^ can be studied (30–35). FLIM *can be performed in the time domain and frequency domain*. FLIM can record fluorescence lifetimes using the time domain technique, time-correlated single-photon counting (TCSPC) (36, 37). An example of retina autofluorescence FLIM is shown in Figure 2 (38).

Fluorescent substances are excited with a short pulse laser, and the time interval it takes for molecules to excite and emit a photon is captured by a detector, allowing the calculation of the fluorescence lifetime. Multiple pulses of light excite the tissue, with the emission of a photon being recorded. The time interval recorded will not always be the same each time a molecule is hit with a light pulse. Given these differences in photon emission behaviors, the lifetimes from each pulse are recorded and sorted by interval duration, which can be graphically depicted on a histogram showing the time-dependent emission of multiple fluorophores in the tissue.

Combining TCSPC-FLIM and two-photon excitation, the generation of three-dimensional imaging of tissue is accomplished (21). This technique allows for acquiring detailed spatial and molecular information from molecule-specific fluorescent lifetimes. Its use has been illustrated in age-related macular degeneration (AMD) and imaging of the retina and choroid (39, 40). One study using two-photon FLIM measured the fluorescence lifetimes in retinal pigment epithelium (RPE) in the porcine retina, showing that the fluorescence lifetimes were relatively short due to melanin fluorescence (41). While fluorescence lifetimes of specific fluorophores can be measured *in vitro* using FLIM, these lifetimes are influenced by various environmental factors, such as pH and oxidation state (17, 20, 42–44). The *in vivo* analysis becomes more challenging due to multiple fluorophores within a tissue sample, complicating the interpretation of fluorescence lifetimes compared to the single-fluorophore analysis possible with *in vitro* FLIM.

**Figure 3:**
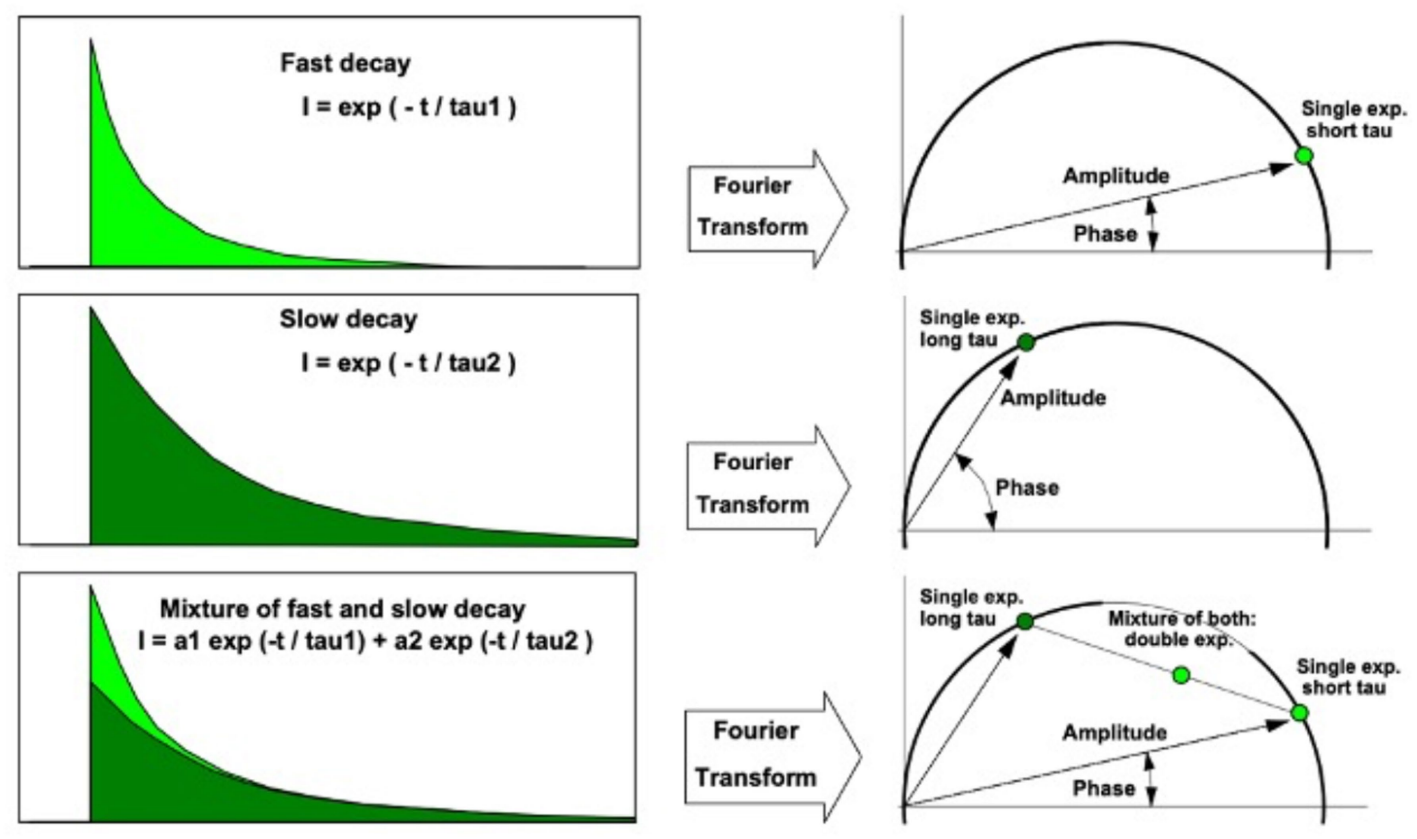
Translation between fluorescence lifetime decay curve (left) and phasor (right). A phasor point directly on the semicircle indicates a single lifetime species. Phasor points of species with multiple fluorescence lifetimes appear inside the semicircle. Adapted from Becker et al. (47).

**Figure 4:**
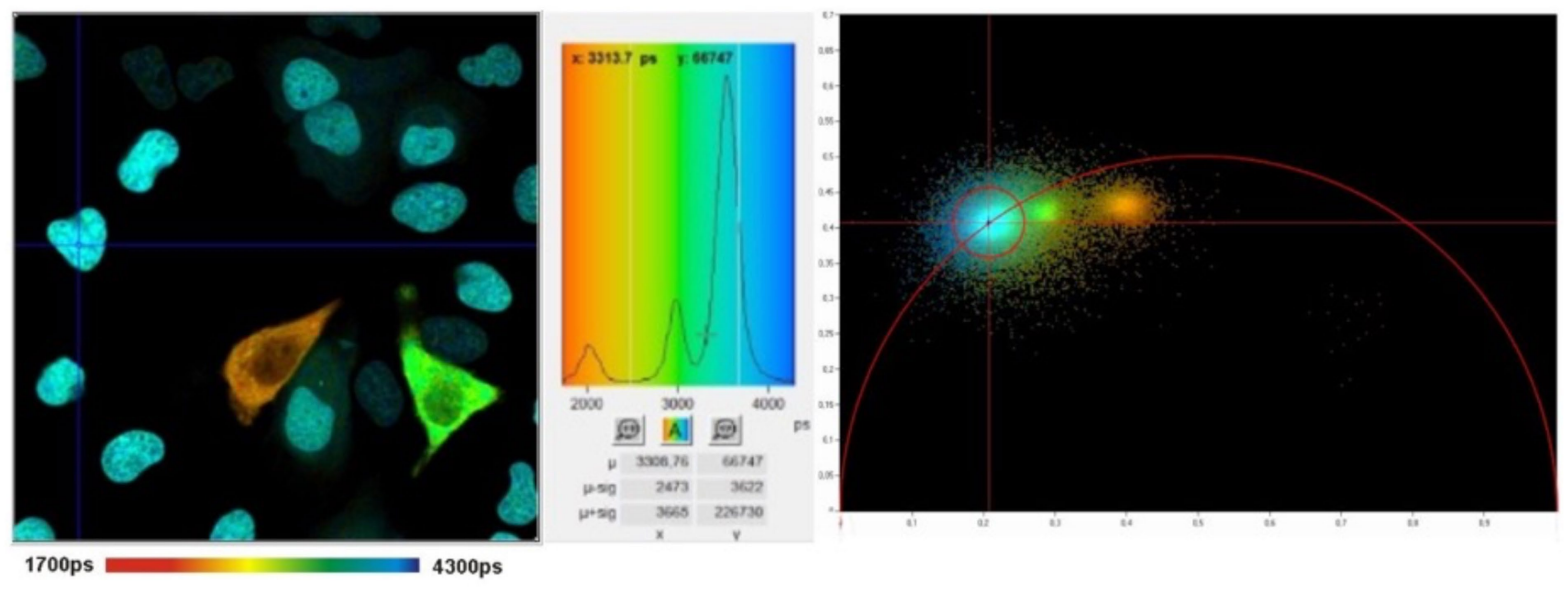
FLIM image (left) with histogram (middle) and corresponding phasor plot (right). The clusters in the phasor plot differentiate pixels of different fluorescence lifetimes in the image. Adapted from Becker et al. (47).

While TCSPC-FLIM techniques plot information on a histogram depicting the time-dependent emission of multiple fluorophores, lifetimes depicted through phasor analysis may be an easier approach for visualizing fluorescence lifetime images of different fluorophores (45, 46). In phasor analysis, fluorescence lifetime data at each pixel can be converted into a coordinate pair known as a phasor (47). Decay data within individual pixels are plotted based on phase and amplitude (47). The phase is defined as the angle of the pointer. While the mathematics of phasor analysis is beyond the scope of this review, the phasor approach uses a rapid Fourier analysis that translates fluorescence lifetime information into a graphical representation called a phasor plot where lifetime differences across various image regions can be distinguished (46). This translation is shown in Figure 3. Phasor plots visualize the representation of the distribution of lifetime values by clustering pixels with similar lifetime properties together in a specific plot area (46). The phasor approach allows for the visual representation of fluorescence lifetimes in entire cells or tissues, providing insight into the state of metabolism or oxidative stress in diseases, as seen in Figure 4 (48). The position of a point on the phasor plot can help differentiate groups of fluorescence lifetimes. For example, lifetimes corresponding to the healthy fovea and macula appear within the semicircle on the shorter-lifetime side (far right side of the phasor plot). In contrast, lifetimes from the optic nerve, which are longer than those from the macula, are located in a distinct cluster on the phasor plot (left of the macula cluster). Phasor plots are currently being incorporated into the analysis of FLIO data.

## 3 Technical aspects of FLIO

FLIO separates itself from FAF intensity imaging by identifying signals dependent on the properties of each fluorophore since each fluorophore has a unique autofluorescence lifetime. In addition, FLIO is not reliant on fluorescence intensity. The differences between FAF intensity imaging and FLIO are shown in Table 1. Current FLIO uses a 473-nm excitation pulsed diode laser, generating pulses at a frequency of 80 MHz with a full width at half maximum of 89 picoseconds (ps). The fiber-coupled laser has an average power of 200 μW, and its safety has been previously studied, fulfilling all safety regulations for a class 1 laser (49, 50).

**Table 1:** Comparison between fundus autofluorescence (FAF) intensity imaging and fluorescence lifetime imaging ophthalmoscopy (FLIO).

Feature	FAF	FLIO
Principle	Measures fluorescence intensity	Measures the time difference from fluorophore excitation to emission (a lifetime)
Use	Assess spatial accumulation of lipofuscin in the retina	SSC: Oxidative metabolism molecules (FAD) LSC: Lipofuscin
Image	Intensity-based grayscale image	Color “heat” map representing different fluorescence lifetimes
Assessment of the retina	Shows where lipofuscin accumulates	Shows the metabolic state of the retina
Time of acquisition per eye	A few seconds	2–3 min

The current system of FLIO measures fluorescence decay across two spectral wavelength channels: a short spectral channel (SSC) ranging from 498 to 560 nm, and a long spectral channel (LSC) ranging from 560 to 720 nm. Although it would be beneficial to obtain FLIO images from other wavelength ranges, the current two-channel system may be practical in clinical settings, as diseases can show changes in one or both spectral channels, providing distinct information about the retina. Additionally, the SSC is somewhat influenced by lens fluorescence, whereas the LSC is relatively unaffected (15, 16, 51). Previous studies have suggested that FAD is detectable within the SSC, while lipofuscin contributes predominantly to the LSC (15, 51, 52). However, these attributions may not fully account for the complexity of *in vivo* fluorescence signals within the retina. Importantly, FAD fluorescence lifetimes are influenced by its role in oxidative metabolism, providing potential insights into mitochondrial activity (21). However, the *in vivo* fluorescence lifetime may be affected by other endogenous fluorophores or changes in the cellular and subcellular environment that share the same emission spectrum as FAD, highlighting the need for further investigation.

Current FLIO technologies record fluorescence lifetimes using the time domain technique, TCSPC (36, 37). FLIO captures images with a resolution of 256 by 256 pixels, covering a 9 by 9 mm area on the retina (35 μm × 35 μm/pixel), which can be focused on the fovea or any other area of the retina (15, 16). The device compensates for eye movements with a high-contrast confocal infrared reflectance image. This technology ensures that each fluorescence photon is accurately registered at its corresponding spatial location on the retina. A minimum signal threshold of approximately 1,000 photons per pixel is used to achieve good-quality FLIO images with an acquisition time of 2–3 min per eye.

**Figure 5:**
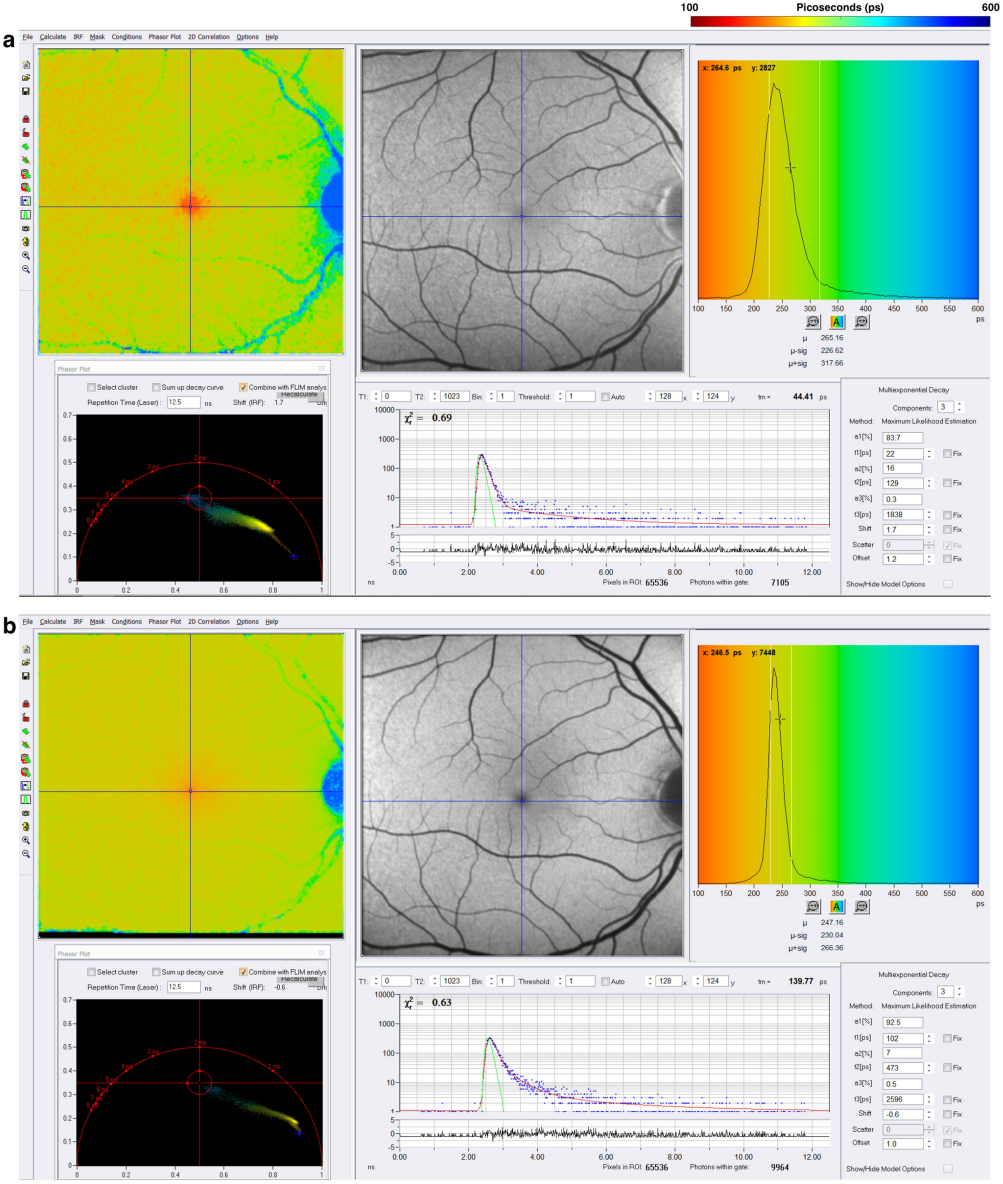
FLIO image of a healthy control after acquisition in the **(A)** short spectral channel (SSC) and **(B)** long spectral channel (LSC). The SSC (498–560 nm) detects fluorescence from multiple fluorophores, including FAD, while the LSC (560–720 nm) captures fluorescence from fluorophores such as lipofuscin. These channels provide information about the metabolic status of the retina.

**Figure 6:**
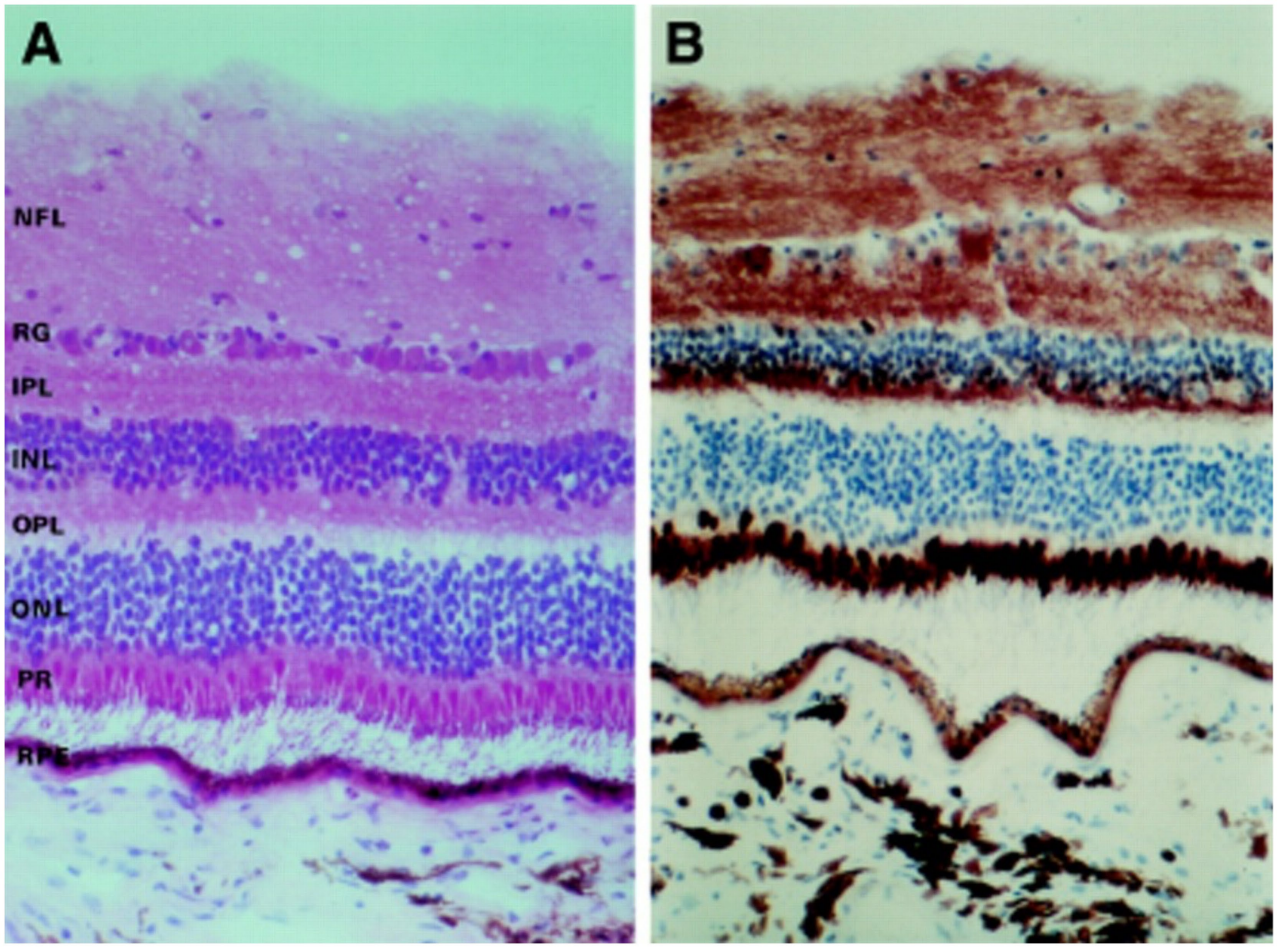
**(A)** Photomicrographs depicting the different retina layers. **(B)** Staining for cytochrome C oxidase, showing the distribution of mitochondria throughout the various layers of the retina. NFL, nerve fiber layer; GC, ganglion cells; IPL, inner plexiform layer; INL, inner nuclear layer; OPL, outer plexiform layer; ONL, outer nuclear layer; PR, photoreceptors; RPE, retinal pigment epithelium. Adapted from Andrews et al. (60).

SPCImage (Becker&Hickl GmbH), the most common software for analyzing FLIO data, uses a triexponential approach to fit the fluorescence decay curve at each pixel (15, 20, 53). During data acquisition, thousands of data points are collected for each pixel, representing the decay of fluorescence over time. The triexponential approach simplifies the data collected by fitting it all into three distinct exponential decay components, each with its own lifetime (τ_1_, τ_2_, τ_3_) and corresponding amplitude, which reflects the relative contribution of individual lifetimes to the total fluorescence decay. At least three points are necessary for the mathematical reconstruction of a decay. The final analysis shows an image where each pixel contains three lifetimes and their relative contributions, which can be visualized in color-coded heat maps. Standardized grids such as the Early Treatment Diabetic Retinopathy Study (ETDRS) grid can segment the images to analyze specific regions (16, 51). FLIO can detect the shortest lifetimes of approximately 30 ps (49). Currently, FLIO-reader and FLIMX are common software packages that can enhance the analysis, image processing, and identification of lifetimes over specific areas of interest within the eye (15). The FLIO output of a healthy retina using SPCImage analysis is shown in Figure 5. In the healthy retina shown in Figure 5, the SSC and LSC histograms display uniform, single peaks, with the SSC exhibiting shorter lifetimes than the LSC. This pattern suggests a more homogeneous distribution of fluorophores contributing to the signal. In contrast, in the conditions discussed later, the peaks in both the SSC and LSC shift toward longer lifetimes and broaden, suggesting increased heterogeneity in the contributing fluorophores (Figure 6).

### 3.1 Fluorescence lifetimes of retinal fluorophores

Previous studies have identified multiple retinal fluorophore properties and fluorescence lifetimes in the context of FLIM (15, 17, 20, 24, 38, 49, 54, 55). FLIO captures fluorescence signals from various retinal layers, including the outer retinal layers, retinal pigment epithelium (RPE), and possibly the choroid (50). The following fluorescence lifetime characteristics of different retinal fluorophores are important in understanding the qualitative and quantitative changes in fluorescence that occur in the retina in various disease settings.

Nicotinamide adenine dinucleotide (NADH) and FAD, important electron carriers in mitochondrial respiration, may impact fluorescence lifetimes because their properties change depending on the mitochondrial metabolic activity of a tissue (56–60). A FAD lifetime depends on its bound state versus free state. Protein-bound FAD has a lifetime of approximately 0.1–0.35 ns, while free FAD has a lifetime of approximately 2.3–2.9 ns due to structural differences in protein-bound and free forms (17, 61–63). A decrease in oxidative metabolism results in a shift toward the free form and so increases the FAD lifetime. FAD fluorescence lifetimes may provide insight into the redox state of the tissue. The emission spectrum of FAD overlaps with the wavelength range of the SSC of FLIO (15, 16).

Lipofuscin fluorescence has been characterized in previous studies and is believed to significantly contribute to retinal autofluorescence due to its brightness (43, 50). In the eye, lipofuscin is mainly found within the RPE and is a product of photoreceptor outer segment degradation (64). Lipofuscin accumulates with age; therefore, it is prevalent in higher amounts in older individuals (53, 65). The main component of lipofuscin, bis-retinoid N-retinyl-N-retinylidene ethanolamine (A2E), has an excitation maximum at 446 nm, an emission maximum at 600 nm, and a mean autofluorescence lifetime of approximately 189 ps (15, 18). Lipofuscin emission is effectively detected by the LSC of FLIO (15, 16). However, multiple overlapping fluorophores may contribute to the fluorescence lifetimes observed in the LSC, and further studies are needed to confirm the specific contributions of lipofuscin and other compounds within the LSC.

Carotenoids, such as lutein, zeaxanthin, and meso-zeaxanthin, comprise the macular pigment (MP) (66, 67). These pigments are found in higher concentrations within the Müller cells and Henle fiber layer of the retina (68, 69). Accumulation of MP within a circular area centered around the fovea is attributed to xanthophyll-binding proteins (70–76). The high amounts of MP serve as antioxidants and help protect the macula from damage, especially in the blue-light range (70, 77, 78). It has been proposed that MP acts to quench free radicals and absorb blue light before it can reach photoreceptors, helping to preserve photoreceptors (69, 79–81). Recent studies using FLIO have demonstrated that the retinal carotenoids of the MP produce a detectable fluorescent signal (49, 51, 70). Unlike FAF intensity imaging, FLIO measures the fluorescence lifetime rather than the intensity of fluorescence. As a result, even carotenoids with weaker fluorescence intensity can still produce a measurable fluorescence lifetime signal.

## 4 FLIO in ophthalmic and neurologic conditions

For comprehensive and in-depth studies of retinal conditions, such as AMD, geographic atrophy, diabetic retinopathy, central serous chorioretinopathy, choroideremia, retinitis pigmentosa, macular holes, and other ophthalmic conditions previously investigated with FLIO, we refer the readers to recent literature (55, 82–97). This review assessed the eyes of healthy individuals in different age groups and selected eye-specific diseases, including macular telangiectasia, Stargardt disease, neuromyelitis optica spectrum disorder, and common neurodegenerative diseases, such as Alzheimer’s disease and Parkinson’s disease.

### 4.1 Healthy eye

In several studies, Schweitzer et al. evaluated the fluorescence lifetimes in healthy eyes (20, 21, 24). Their first device used an excitation laser of 446 nm and collected fluorescence lifetime information of a 25-year-old healthy adult in two spectral channels: 510–560 nm and 560–700 nm. Their study showed short fluorescence lifetimes at the macula (150 ps) and longest lifetimes near the optic disc (250 ps) (20). A study by Dysli et al. in healthy eyes later identified a similar pattern of fluorescence lifetimes, showing the shortest mean fluorescence lifetimes at the macula (SSC: 208 ps; LSC: 239 ps), with increased lifetimes further away from the center of the retina (53). Their study investigated a group of patients with a mean age of 35 years. Fluorescence lifetimes were obtained within the ETDRS grid. The longest lifetimes were found at the optic disc, consistent with a previous study by Schweitzer et al. Sauer et al. investigated the fluorescence lifetimes of patients with an average age of 24 years, identifying the shortest lifetimes occurring at the fovea (SSC: 82 ps; LSC: 126 ps) (49). The differences in values of fluorescence lifetimes can be attributed to using a biexponential approach in the study by Dysli et al. and a triexponential approach by Sauer et al. (49, 53). In these studies, lifetimes appear to prolong with age in both the SSC and LSC, but especially within the LSC; this pattern is consistent with the accumulation of lipofuscin with age, which prolongs lifetimes recorded in the LSC, where lipofuscin is primarily detectable (20, 49, 53).

**Figure 7:**
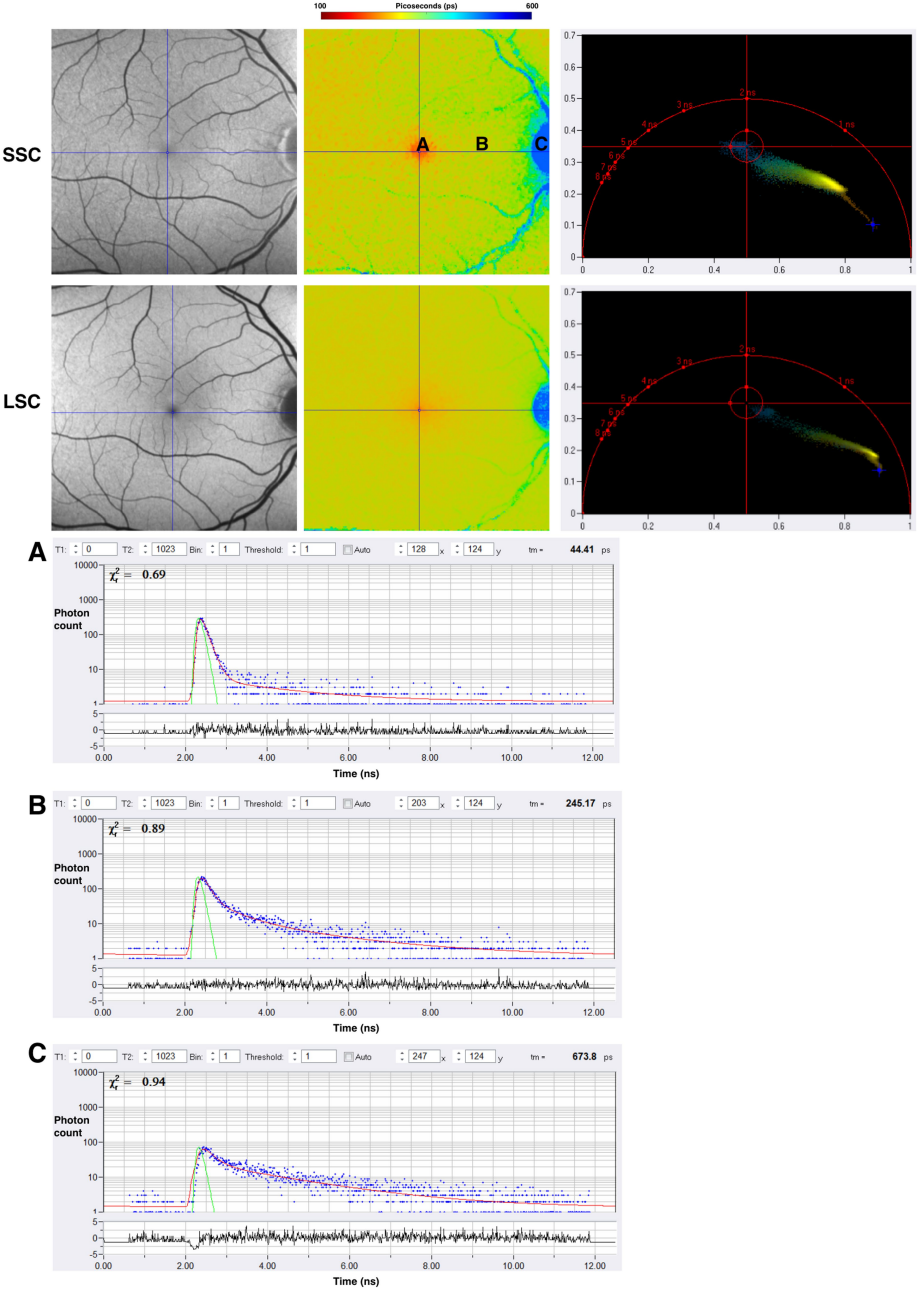
FAF intensity and FLIO images of the healthy eye. Both the SSC (498–560 nm) and LSC (560–720 nm) are shown. **(A–C)** Are individual pixels with lifetime decay curves shown. Phasor plots for SSC and LSC are shown as well.

Across previously described studies assessing the fluorescence lifetimes of healthy eyes, the shortest lifetimes appear to be at the foveal and macular regions, with the longest lifetimes appearing at the optic disc. Figure 7 displays the lifetime patterns seen in healthy eyes. Studies have suggested that the long lifetimes detected by FLIO within the LSC at the optic disc are primarily due to the higher concentration of connective tissue, such as collagen and elastin, within the optic disc (20). The shortest lifetimes are located at the fovea and central macula, likely due to the influence of macular pigment (MP) and carotenoids (15, 51). As previously mentioned, in the early 2000s, it was hypothesized that MP did not emit fluorescence but could only absorb it. However, subsequent studies showed that MP can emit fluorescence, with later FLIO studies identifying a correlation between MP and short foveal fluorescence lifetimes (51, 82, 98). The contribution of various retinal molecules correlates to different patterns of fluorescence lifetimes across different areas of the retina. These patterns later helped identify changes in fluorescence lifetimes in different ophthalmic and neurologic diseases.

### 4.2 Macular telangiectasia type 2 (MacTel)

Macular Telangiectasia Type 2, an inherited disease, causes bilateral central vision loss, usually between the fourth and sixth decades of life (16, 99). While classically presenting later in life, some case reports have reported patients being diagnosed in their 20s (100). MacTel is primarily a retinal degenerative disease affecting the Müller Cells with secondary vascular changes. Visual loss progresses slowly, with patients frequently reporting problems with reading and experiencing diminished visual acuity (101). Structurally, MacTel has been described as degeneration of the ellipsoid zone (EZ) starting in the temporal parafoveal area (101). Around the fovea, a characteristic pattern is seen in MacTel with FLIO. This pattern is an oval-shaped area, 9 degrees horizontal and 5 degrees vertical, centered at the fovea (100, 101). This area with FLIO shows a ring or crescent shape of prolonged lifetimes (100). In one study investigating the use of FLIO in MacTel, researchers found prolonged lifetimes of the inner temporal area on the ETDRS grid in MacTel patients (SSC: 382 ps) when compared to control, healthy eyes (SSC: 298 ps) (100).

In another study, FLIO was used to investigate early changes in retinal disease in the children of MacTel patients (102). This study found the characteristic temporal parafoveal prolonged lifetimes within the SSC in MacTel patients and in over one-third of unaffected children of these MacTel patients (102). As MacTel is a progressive disease, studies have investigated the speed of progression of the disease as it correlates to FLIO lifetimes. One study found a 22% prolongation of SSC lifetimes over 2.1 years in a group of four patients with MacTel (103). Another study identified 33 patients with MacTel and found an annual progression of 9 ps in the SSC and 8 ps in the LSC lifetimes, compared to controls (101). A more recent study identifying 49 eyes with MacTel confirmed the unique temporal crescent pattern of prolonged fluorescence lifetimes around the fovea, especially in the SSC, while other retinal diseases tend to prolong lifetimes in the LSC (104). In one study investigating cases of MacTel, FLIO demonstrated a sensitivity of 96% and a specificity of 100% (105). The findings from studies investigating MacTel reiterate the benefit of FLIO as an accurate diagnostic tool and a potential screening tool in early disease detection before patients experience visual changes. Early detection may aid in the advancement of future therapies and gene investigation.

### 4.3 Stargardt disease

**Figure 8:**
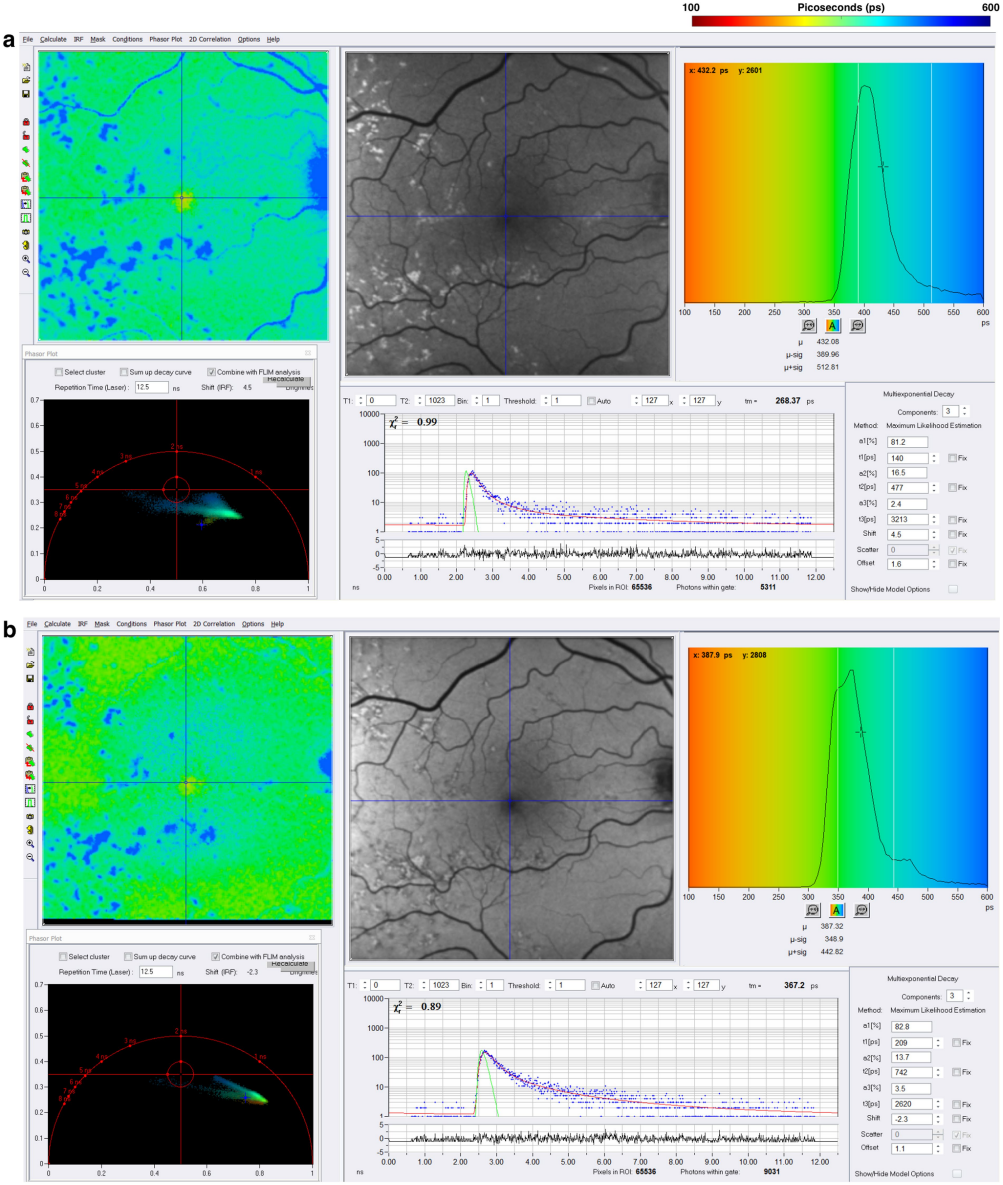
FLIO images from a patient with a long history of Stargardt disease with corresponding phasor plots. Images of both **(A)** SSC and **(B)** LSC are shown. The FLIO images show blue “flecks” that have been present for a longer period (113). Yellow “flecks” are not presented here as they are present in the acute phase of Stargardt disease (113).

Stargardt disease, an inherited retinal dystrophy, usually presents in childhood with bilateral severe vision loss (106–108). Mutations in the ABCA4 gene, which codes for an ATP-binding cassette protein that aids in transporting N-retinylidene-phosphatidylethanolamine from the photoreceptor disk lumen to the cytoplasm, form the genetic basis for this disease (108). Visual cycle molecules such as bis-retinoids and lipofuscin accumulate in this disease (109–111). FAF intensity imaging shows increased signal even in areas with seemingly normal functioning photoreceptors (111). The increase in FAF intensity is attributed to the accumulation of lipofuscin in the RPE rather than direct dysfunction of the photoreceptors themselves (110–113). As the disease progresses, yellow spots appear on the retina, termed retinal “flecks” (113). Retinal flecks are thought to be areas of retinal degeneration (113). In the study of 16 patients with Stargardt disease, Dysli et al. found that the retinal flecks showed both short and prolonged FLIO lifetimes depending on the age of the flecks (112). Retinal flecks with short (242 ps) lifetimes in the LSC were believed to be new onset flecks attributed to compounds of degenerating photoreceptors, while flecks with long (474 ps) lifetimes were presumed to be flecks present for longer periods, influenced by lipofuscin and A2E (112). Throughout the disease, there is a gradual progression from short to long FLIO lifetimes in the LSC (113). In Stargardt disease, FLIO shows potential in monitoring disease progression and may eventually track treatment responses. A representative example of a patient with Stargardt disease is shown in Figure 8.

### 4.4 Alzheimer’s disease (AD)

**Figure 9:**
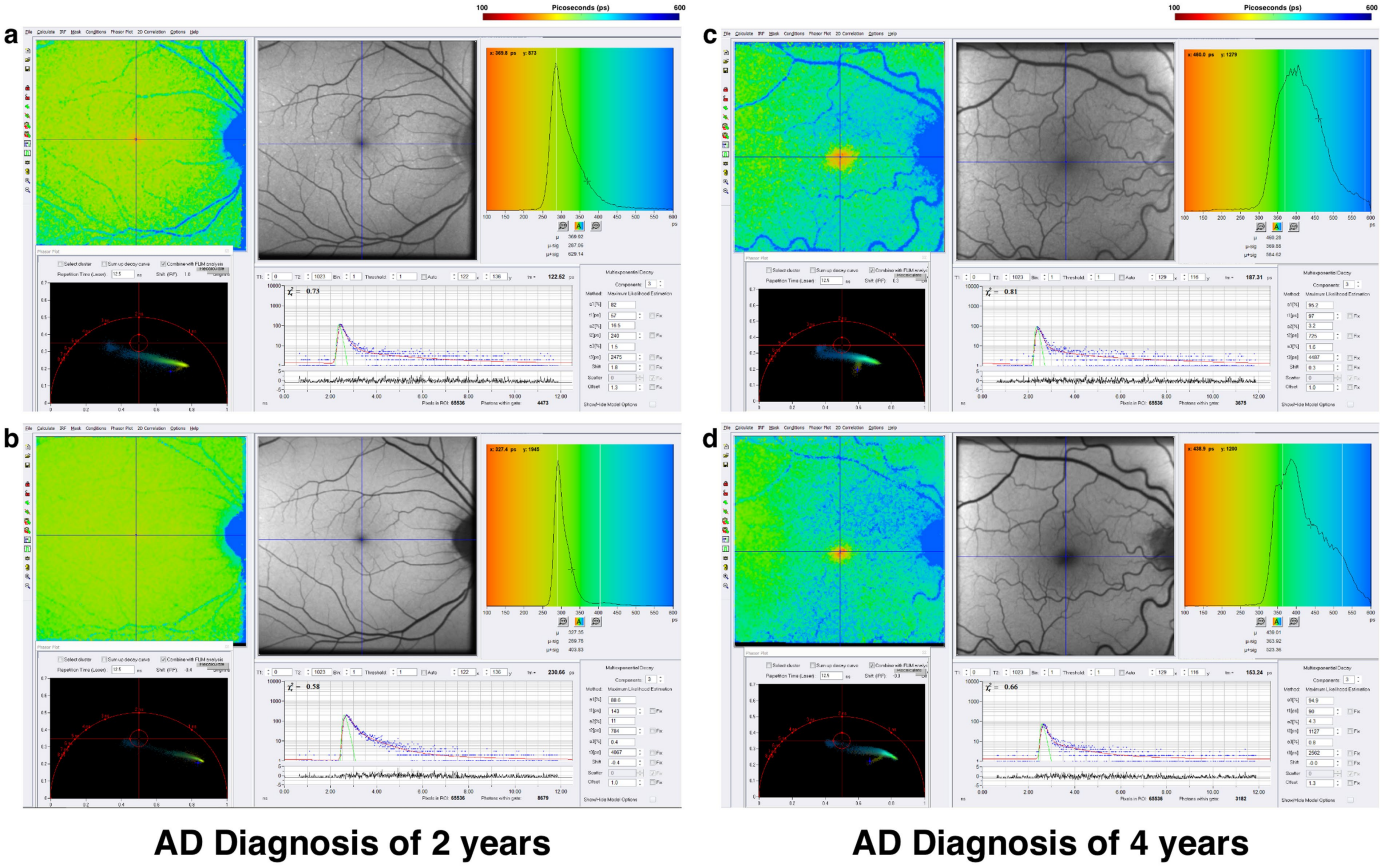
FLIO images and phasor plots of a patient with a 2-year diagnosis of AD showing both the **(A)** SSC and **(B)** LSC. Also shown is a patient with a 4-year diagnosis of AD showing both the **(C)** SSC and **(D)** LSC.

The rationale to study FLIO in AD evolved from the importance of vision changes and the retina’s role as an extension of the central nervous system, as intriguing OCT and FAF data have shown retinal degeneration and retinal nerve fiber layer thinning (114–117). Jentsch et al. investigated the use of FLIO in 16 patients with AD finding FLIO parameters of amplitudes (*α*) and relative contributions (Q) within the LSC correlated significantly with the mini-mental status exam score (Q_2_, *R* = −0.757, *p* = 0.007; α_2_, *R* = −0.618, *p* = 0.043), as well as p-tau-181 concentration in CSF (Q_2_, *R* = 0.919, *p* = 0.009; α_2_, *R* = 0.881, *p* = 0.020) (118). While FLIO lifetimes were not necessarily prolonged, the changes identified in this study suggest a potential role for FLIO as a valuable tool in the early diagnosis of AD-associated changes within the retina (118). Another pilot study investigated FLIO in patients with preclinical AD (7 AD, 8 control) (119). In phakic patients, investigators showed that patients with AD, when compared to controls, had significantly prolonged mean fluorescence lifetimes within the SSC (AD: 593.9 ps; control: 475.0 ps; *p* = 0.036) and LSC (AD: 454.4 ps; control: 394.1 ps; *p* = 0.024) (119). They also found that amyloid *β*, tau in CSF, and ganglion cell layer plus inner plexiform layer thickness (as determined by OCT) were correlated with mean fluorescence lifetimes in phakic subjects (*r* = −0.611–0.562, *p* < 0.05) (119). A recent study presented at the 2024 Alzheimer’s Association International Conference showed that patients with AD (11 patients) demonstrated prolonged lifetimes in both the SSC and LSC when compared to controls (11 patients) (120). Although these studies had a limited number of patients, the initial findings suggest that FLIO may detect fluorophores linked to mitochondrial metabolic impairments in AD, pending further investigation to accurately identify the source of these signals. Representative examples of patients with AD are shown in Figure 9.

### 4.5 Parkinson’s disease (PD)

Parkinson’s disease has many motor and non-motor manifestations, especially in the visual system, including abnormalities in eye movements, visual acuity, color vision, and contrast sensitivity (121–123). Mitochondrial pathogenic factors have also been identified in the initiation and progression of PD. (124–126). Multiple studies investigating OCT in PD have shown retinal nuclear layer thinning, reduced retinal nerve fiber layer, and reduced macular volume (127, 128). While OCT helps to assess structural changes in PD after they have already occurred, understanding early metabolic changes may help identify the disease before the clinical onset of visual changes.

In a pilot study presented at the 2023 International Congress of Parkinson’s Disease and Movement Disorders, Shivok et al. showed nine PD patients experiencing visual disturbances and compared them to nine controls (129). Their data showed that PD patients had prolonged mean fluorescence lifetimes within the SSC in the right eye and both SSC and LSC in the left eye when compared to controls (129). At the 2024 ARVO annual meeting, another study showed that lifetimes were prolonged within the SSC of PD patients (19 patients without apparent retinal disease) when compared to controls (20 patients) (130). At the 2024 International Congress of Parkinson’s Disease and Movement Disorders, investigators showed that 27 PD patients had prolonged mean fluorescence lifetimes in both the SSC and LSC compared to 9 controls (131).

**Figure 10:**
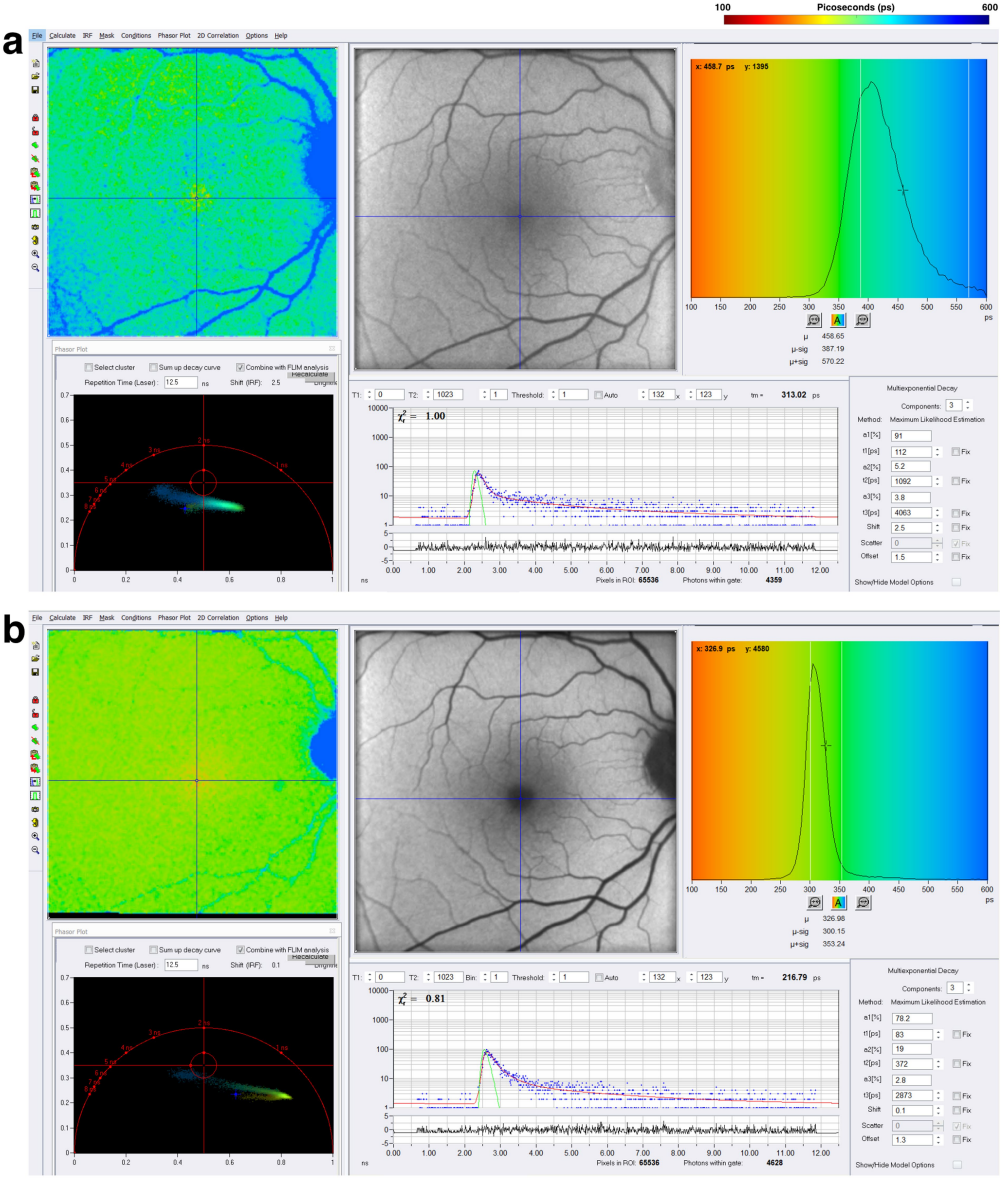
FLIO images and phasor plots of a patient with PD. Both **(A)** SSC and **(B)** LSC are shown.

These initial FLIO pilot studies observed prolonged fluorescence lifetimes in PD patients, suggesting complex *in vivo* metabolic changes in the retina. The longer FLIO lifetimes seen in patients with PD may be influenced by the cumulative impact of neurodegenerative changes, oxidative stress, or other mechanisms. Further studies with FLIO are needed to show that metabolic changes may occur, and these changes may occur before the onset of visual symptoms associated with the disease. A representative example of a patient with PD is shown in Figure 10.

### 4.6 Neuromyelitis optica spectrum disorder (NMOSD)

NMOSD, an antibody-mediated inflammatory disease directed against aquaporin 4 (AQP4) water channels, may result in severe vision loss. AQP4 has been shown to co-localize and interact with inwardly rectifying potassium channels within the retina (132). These potassium channels aid in spatial buffering of retinal potassium, a process mediated by retinal astrocytes (Müller cells) (132–134). Müller cells assist in the balance of fluid movement within the retina and span from the inner retina to the inner segments of the photoreceptors. Müller cell footplates have high concentrations of AQP4 channels that co-localize with potassium channels and AQP4 channels scattered on the surface of Müller cells (132). The Müller cell foot processes surround retinal vessels in the superficial and deep vascular layers of the retina (132). In NMOSD, these interactions are disrupted, leading to retinal swelling and edema, potentially resulting in permanent visual loss (135). These structural retinal changes have traditionally been visualized with OCT, but FLIO provides insight into the metabolic changes occurring within the retina in patients with NMOSD.

Recent studies have suggested that mitochondrial dysfunction contributes to the pathophysiology of NMOSD (136, 137). Mitochondrial dysfunction has been implicated in motor and cognitive symptoms of NMOSD (136). In an animal model of NMOSD, researchers showed that etomoxir, a drug that interferes with mitochondrial fatty acid oxidation, can modulate mitochondrial function and ameliorate astrocyte pathology associated with NMOSD (137). While astrocytes with mitochondrial dysfunction can still survive, they lose their neuroprotective functions, a crucial factor in NMOSD (137). Their study suggests that mitochondrial dysfunction in astrocytes and retinal Müller cells may exacerbate retinal swelling and visual damage from disruptions in metabolic activity and oxidative stress.

**Figure 11:**
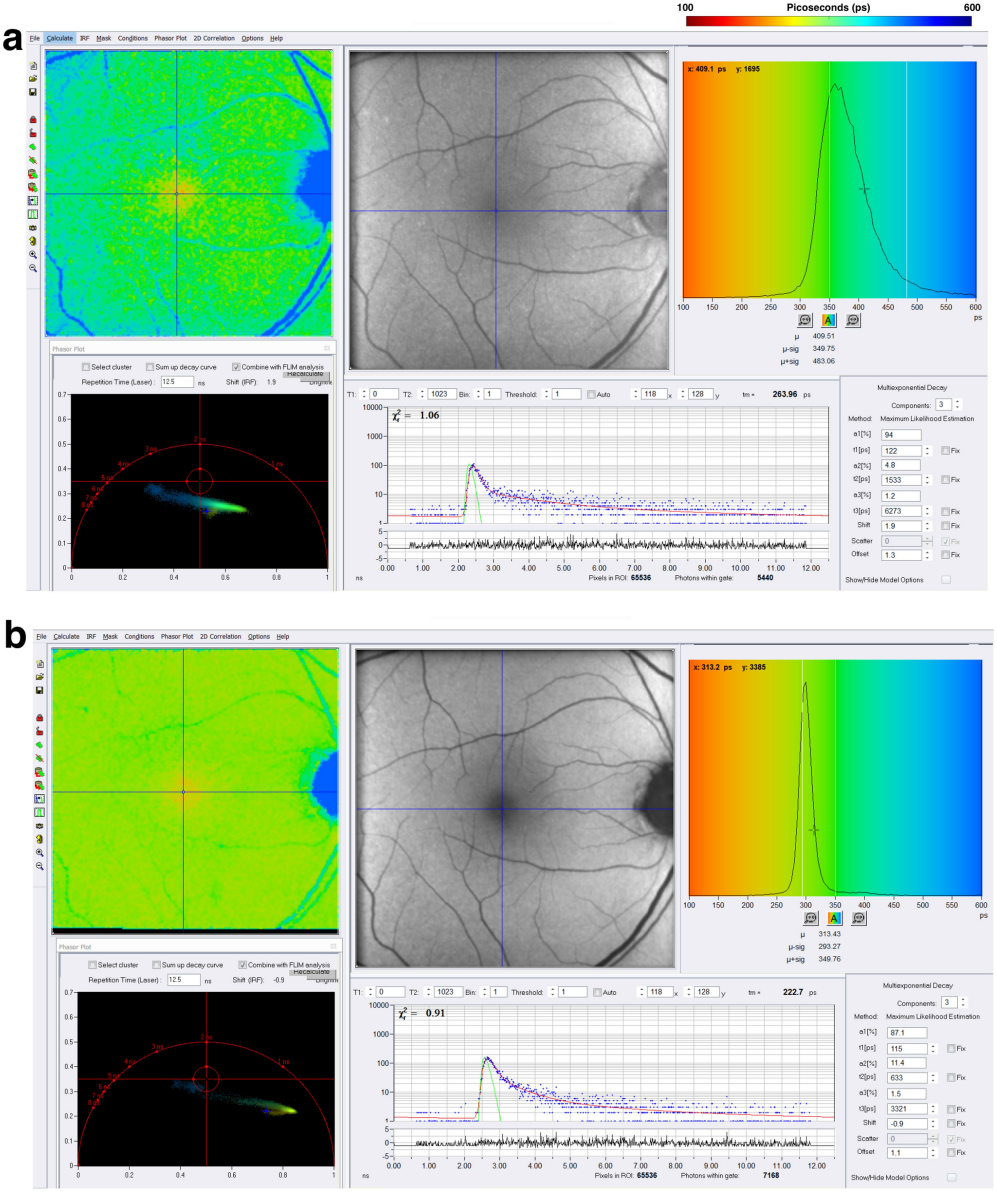
FLIO images and phasor plots of a patient with NMOSD. Both **(A)** SSC and **(B)** LSC are shown.

In a pilot study, Cappellani et al. investigated FLIO in nine patients (18 eyes) with NMOSD and compared them with 12 controls (24 eyes) (138). The study found significantly prolonged mean FLIO lifetimes (τ_m_) in the SSC in patients with NMOSD compared to controls (NMOSD: 181.71 ps, controls: 118.46 ps, *p* = 0.004) (138). OCT showed significant differences in retinal nerve fiber layer (RNFL) thickness average (NMOSD: 80.5 μm, controls: 96.2 μm, *p* = 0.046), ganglion cell layer (GCL) volume (NMOSD: 0.86 mm^3^, controls: 1.07 mm^3^, *p* = 0.0009), and GCL thickness at the 3 mm nasal area (NMOSD: 37.1 μm, controls: 53.2 μm, *p* = 0.000245) (138). The pilot study suggested that both structural and functional changes are seen in NMOSD, with functional changes detected by FLIO and structural changes seen on OCT, specifically in the GCL. Given that the GCL is an area highly concentrated in AQP4 channels at the retinal capillary-Müller cell junction, supplied from the central retinal artery, these findings suggest that central retinal artery ischemic changes to Müller cells contribute to metabolic dysfunction and permanent visual loss in NMOSD (138). A representative example of NMOSD is shown in Figure 11.

## 5 Summary and conclusion

1. FLIO provides a unique, non-invasive method with the potential to monitor fluorescence signals linked to the metabolic state of the retina and to reveal their alterations in various ophthalmic and neurologic conditions. Acquiring data with FLIO is fast and may be effective in a clinical setting.
2. Deciphering FLIO data with phasor plots in the clinical setting may allow for the rapid and more specific interpretation of data, aiding in the diagnosis and assessment of disease. Correlating FLIO findings with OCT might provide qualitative and quantitative data on the structural and metabolic state of the retina in various diseases, potentially improving personalized disease management.
3. Upon validation of the molecular components of FLIO data, which can be achieved by complementing FLIM studies, FLIO could allow for understanding the metabolic state of specific fluorophores within the retina. Future advancements in two-photon excitation FLIO can potentially expand the depth and scope of information beyond what the current setup of FLIO offers, enabling the detection of other important molecules such as NADH. The current understanding of FLIO requires further investigation through molecular imaging and pharmacological validation to accurately link fluorescence lifetimes to specific retinal molecules *in vivo*.
4. The use of FLIO in MacTel and Stargardt disease shows early potential to document metabolic changes before symptoms of vision change begin, aiding in the early detection of disease. Thus, FLIO technology has shown potential in detecting functional changes from the studies outlined in this review, sometimes preceding structural changes observed by traditional imaging modalities.
5. Based upon preliminary data, FLIO may have a role in the early detection of broad neurodegenerative conditions such as AD and PD and may also be useful in other conditions such as neuroinflammation exemplified by NMOSD.
6. The retina provides at least a window, and maybe a microcosm, into the central nervous system, and the use of FLIO in neurology and neuro-ophthalmology is increasing.
7. Classifying diseases based on abnormal FLIO signals requires disease-specific pattern recognition in fluorescence lifetimes. These patterns can be qualitative, as demonstrated by the distinct spatial patterns of lifetimes observed in Stargardt disease and MacTel type 2, which can be differentiated from one another based on their unique FLIO findings. These patterns can also be quantitative, such as the prolonged lifetimes observed in many conditions discussed in this review. For example, FLIO can already help differentiate new flecks from older flecks in Stargardt disease (112, 113). In the future, the spatial distribution of fluorescence lifetimes across different retinal layers may further aid in classifying disease, as many diseases affect specific layers of the retina differently. Additionally, the use of FLIO technology with multiple excitation and emission wavelengths in the future may help to establish disease-specific patterns of fluorescence lifetimes by capturing a broader range of fluorophore contributions.
8. Future strategies may involve developing disease-specific reference databases and using artificial intelligence (AI) to analyze fluorescence lifetime images, histograms, and phasor plots, enabling automated detection of disease-related abnormalities that are diagnostically accurate and reproducible. Additionally, integrating FLIO with imaging modalities, such as OCT, could improve disease classification by correlating metabolic abnormalities with retinal layer-specific structural changes. Recent advancements in FLIO analysis include the integration of AI, such as support vector machines, which have successfully differentiated fluorescence lifetimes between non-smokers and heavy smokers with an accuracy of 80% (139). Adaptive optics fluorescence lifetime imaging ophthalmoscopy (AOFLIO) is a technical enhancement of FLIO, acquiring fluorescence lifetimes with higher resolution and primarily from the RPE (140). This advancement of FLIO found prolonged lifetimes in patients with pentosan polysulfate toxicity, emphasizing subtle outer retina changes that are not visible with standard imaging modalities (140). Near-infrared AOFLIO is another technique that has enhanced standard FLIO technology to visualize the RPE cellular mosaic (141). These advancements highlight the potential of AI-assisted and AOFLIO techniques to enhance diagnostic accuracy and expand FLIO in ophthalmic and neurodegenerative diseases. FLIO has the potential to become a valuable tool for the early detection and monitoring of disease progression and for measuring the impact of experimental therapies on disease.

## References

[1] Arnold AC Costa RM Dumitrascu OM. The spectrum of optic disc ischemia in patients younger than 50 years (an Amercian ophthalmological society thesis). Trans Am Ophthalmol Soc. (2013) 111:93–118. PMID: 24167327 PMC3799463

[2] Arnold AC Hepler RS. Fluorescein angiography in acute nonarteritic anterior ischemic optic neuropathy. Am J Ophthalmol. (1994) 117:222–30. doi: 10.1016/S0002-9394(14)73080-6, PMID: 8116751

[3] How AC Koh AH. Angiographic characteristics of acute central serous chorioretinopathy in an Asian population. Ann Acad Med Singap. (2006) 35:77–9. doi: 10.47102/annals-acadmedsg.V35N2p77, PMID: 16565758

[4] Kim MK Kim US. Analysis of fundus photography and fluorescein angiography in Nonarteritic anterior ischemic optic neuropathy and optic neuritis. Korean J Ophthalmol. (2016) 30:289–94. doi: 10.3341/kjo.2016.30.4.289, PMID: 27478356 PMC4965604

[5] Littlewood R Mollan SP Pepper IM Hickman SJ. The utility of fundus fluorescein angiography in neuro-ophthalmology. Neuroophthalmology. (2019) 43:217–34. doi: 10.1080/01658107.2019.1604764, PMID: 31528186 PMC6736131

[6] Marmor MF Ravin JG. Fluorescein angiography: insight and serendipity a half century ago. Arch Ophthalmol. (2011) 129:943–8. doi: 10.1001/archophthalmol.2011.160, PMID: 21746986

[7] Mishra C Tripathy K. Fundus camera. Treasure Island: StatPearls (2024). 36256758

[8] Perez MA Bruce BB Newman NJ Biousse V. The use of retinal photography in nonophthalmic settings and its potential for neurology. Neurologist. (2012) 18:350–5. doi: 10.1097/NRL.0b013e318272f7d7, PMID: 23114666 PMC3521530

[9] Ruia S Tripathy K. Fluorescein angiography Treasure Island (FL), StatPearls (2024). 35015403

[10] Pujari A Bhaskaran K Sharma P Singh P Phuljhele S Saxena R. Optical coherence tomography angiography in neuro-ophthalmology: current clinical role and future perspectives. Surv Ophthalmol. (2021) 66:471–81. doi: 10.1016/j.survophthal.2020.10.009, PMID: 33157113

[11] Minakaran N de Carvalho ER Petzold A Wong SH. Optical coherence tomography (OCT) in neuro-ophthalmology. Eye. (2021) 35:17–32. doi: 10.1038/s41433-020-01288-x, PMID: 33239763 PMC7852683

[12] Murthy RK Haji S Sambhav K Grover S Chalam KV. Clinical applications of spectral domain optical coherence tomography in retinal diseases. Biom J. (2016) 39:107–20. doi: 10.1016/j.bj.2016.04.003, PMID: 27372166 PMC6138795

[13] Greenberg BM Frohman E. Optical coherence tomography as a potential readout in clinical trials. Ther Adv Neurol Disord. (2010) 3:153–60. doi: 10.1177/1756285610368890, PMID: 21179607 PMC3002650

[14] Spaide RF Fujimoto JG Waheed NK Sadda SR Staurenghi G. Optical coherence tomography angiography. Prog Retin Eye Res. (2018) 64:1–55. doi: 10.1016/j.preteyeres.2017.11.003, PMID: 29229445 PMC6404988

[15] Sauer L Andersen KM Dysli C Zinkernagel MS Bernstein PS Hammer M. Review of clinical approaches in fluorescence lifetime imaging ophthalmoscopy. J Biomed Opt. (2018) 23:1–20. doi: 10.1117/1.JBO.23.9.091415, PMID: 30182580 PMC8357196

[16] Sauer L Vitale AS Modersitzki NK Bernstein PS. Fluorescence lifetime imaging ophthalmoscopy: autofluorescence imaging and beyond. Eye. (2021) 35:93–109. doi: 10.1038/s41433-020-01287-y, PMID: 33268846 PMC7852552

[17] Dysli C Wolf S Berezin MY Sauer L Hammer M Zinkernagel MS. Fluorescence lifetime imaging ophthalmoscopy. Prog Retin Eye Res. (2017) 60:120–43. doi: 10.1016/j.preteyeres.2017.06.005, PMID: 28673870 PMC7396320

[18] Lakowicz JR. Principles of fluorescence spectroscopy. 3rd ed. New York: Springer (2006). 954 p.

[19] Chen LC Lloyd WR III Chang CW Sud D Mycek MA. Fluorescence lifetime imaging microscopy for quantitative biological imaging. Methods Cell Biol. (2013) 114:457–88. doi: 10.1016/B978-0-12-407761-4.00020-8, PMID: 23931519

[20] Schweitzer D Schenke S Hammer M Schweitzer F Jentsch S Birckner E. Towards metabolic mapping of the human retina. Microsc Res Tech. (2007) 70:410–9. doi: 10.1002/jemt.20427, PMID: 17393496

[21] Schweitzer D Kolb A Hammer M Anders R. Time-correlated measurement of autofluorescence. A method to detect metabolic changes in the fundus. Ophthalmologe. (2002) 99:774–9. doi: 10.1007/s00347-002-0656-3, PMID: 12376853

[22] Schweitzer D Kolb A Hammer M Thamm E. Basic investigations for 2-dimensional time-resolved fluorescence measurements at the fundus. Int Ophthalmol. (2001) 23:399–404. doi: 10.1023/A:1014475219117, PMID: 11944867

[23] Schweitzer D Hammer M Anders R Doebbecke T. Evaluation of time-resolved autofluorescence images of the ocular fundus In: Wagnieres GA editor. Diagnostic Optical Spectroscopy in Biomedicine II. Munich: Optica Publishing Group (2003).

[24] Schweitzer D Hammer M Schweitzer F Anders R Doebbecke T Schenke S. In vivo measurement of time-resolved autofluorescence at the human fundus. J Biomed Opt. (2004) 9:1214–22. doi: 10.1117/1.1806833, PMID: 15568942

[25] Schweitzer D Hammer M Jentsch S Schenke S. Interpretation of measurements of dynamic fluorescence of the eye. Optics East. (2007) 6771:1–12. doi: 10.1117/12.735815

[26] Galletly NP McGinty J Dunsby C Teixeira F Requejo-Isidro J Munro I. Fluorescence lifetime imaging distinguishes basal cell carcinoma from surrounding uninvolved skin. Br J Dermatol. (2008) 159:152–61. doi: 10.1111/j.1365-2133.2008.08577.x, PMID: 18460029

[27] Phipps J. Fluorescence lifetime imaging microscopy for the characterization of atherosclerotic plaques. Proc SPIE Int Soc Opt Eng. (2009) 7161:71612G. doi: 10.1117/12.813087 PMC2709872 19606277

[28] Marcu L. Fluorescence lifetime in cardiovascular diagnostics. J Biomed Opt. (2010) 15:011106. doi: 10.1117/1.3327279, PMID: 20210432 PMC2847934

[29] Bec J Xie H Yankelevich DR Zhou F Sun Y Ghata N. Design, construction, and validation of a rotary multifunctional intravascular diagnostic catheter combining multispectral fluorescence lifetime imaging and intravascular ultrasound. J Biomed Opt. (2012) 17:106012. doi: 10.1117/1.JBO.17.10.106012, PMID: 23224011 PMC3461057

[30] Wu Z Liu M Liu Z Tian Y. Real-time imaging and simultaneous quantification of mitochondrial H(2)O(2) and ATP in neurons with a single two-photon fluorescence-lifetime-based probe. J Am Chem Soc. (2020) 142:7532–41. doi: 10.1021/jacs.0c00771, PMID: 32233469

[31] Lakowicz JR Szmacinski H Johnson ML. Calcium imaging using fluorescence lifetimes and long-wavelength probes. J Fluoresc. (1992) 2:47–62. doi: 10.1007/BF00866388, PMID: 24243158 PMC6885754

[32] Datta R Gillette A Stefely M Skala MC. Recent innovations in fluorescence lifetime imaging microscopy for biology and medicine. J Biomed Opt. (2021) 26:070603. doi: 10.1117/1.JBO.26.7.070603, PMID: 34247457 PMC8271181

[33] Csordás G Várnai P Golenár T Roy S Purkins G Schneider TG. Imaging interorganelle contacts and local calcium dynamics at the ER-mitochondrial interface. Mol Cell. (2010) 39:121–32. doi: 10.1016/j.molcel.2010.06.029, PMID: 20603080 PMC3178184

[34] Lakowicz JR Szmacinski H. Fluorescence lifetime-based sensing of pH, Ca2+, K+ and glucose. Sensors Actuators B Chem. (1993) 11:133–43. doi: 10.1016/0925-4005(93)85248-9 PMC8049533 33867678

[35] Datta R Heaster TM Sharick JT Gillette AA Skala MC. Fluorescence lifetime imaging microscopy: fundamentals and advances in instrumentation, analysis, and applications. J Biomed Opt. (2020) 25:1–43. doi: 10.1117/1.JBO.25.7.071203, PMID: 32406215 PMC7219965

[36] Becker W. The bh TCSPC handbook. 10th ed. Becker and Hickl GmbH. (2023).

[37] Becker W Bergmann A Hink MA König K Benndorf K Biskup C. Fluorescence lifetime imaging by time-correlated single-photon counting. Microsc Res Tech. (2004) 63:58–66. doi: 10.1002/jemt.10421, PMID: 14677134

[38] Kesavamoorthy N Junge JA Fraser SE Ameri H. Insights into metabolic activity and structure of the retina through multiphoton fluorescence lifetime imaging microscopy in mice. Cells. (2022) 11:2265. doi: 10.3390/cells11152265, PMID: 35892562 PMC9331481

[39] Han M Giese G Schmitz-Valckenberg S Bindewald-Wittich A Holz FG Yu J. Age-related structural abnormalities in the human retina-choroid complex revealed by two-photon excited autofluorescence imaging. J Biomed Opt. (2007) 12:024012. doi: 10.1117/1.2717522, PMID: 17477727

[40] La Schiazza O Bille JF. High-speed two-photon excited autofluorescence imaging of ex vivo human retinal pigment epithelial cells toward age-related macular degeneration diagnostic. J Biomed Opt. (2008) 13:064008. doi: 10.1117/1.2999607 19123655

[41] Peters S Hammer M Schweitzer D. Two-photon excited fluorescence microscopy application for ex vivo investigation of ocular fundus samples In: Peter TC Emmanuel B editors. Advanced microscopy techniques II. Munich: Optica Publishing Group (2011).

[42] Suhling K French PM Phillips D. Time-resolved fluorescence microscopy. Photochem Photobiol Sci. (2005) 4:13–22. doi: 10.1039/b412924p, PMID: 15616687

[43] Delori FC Dorey CK Staurenghi G Arend O Goger DG Weiter JJ. In vivo fluorescence of the ocular fundus exhibits retinal pigment epithelium lipofuscin characteristics. Invest Ophthalmol Vis Sci. (1995) 36:718–29. 7890502

[44] Schweitzer D Jentsch S Schenke S Hammer M Biskup C Gaillard E. Spectral and time-resolved studies on ocular structures. Eur Conf Biomed Optics. SPIE BiOS. (2007) 6628:1–12. doi: 10.1117/12.726701

[45] Digman MA Caiolfa VR Zamai M Gratton E. The phasor approach to fluorescence lifetime imaging analysis. Biophys J. (2008) 94:L14–6. doi: 10.1529/biophysj.107.120154, PMID: 17981902 PMC2157251

[46] Colyer R Siegmund O Colyer R Colyer R Colyer R Colyer R. Phasor-based single-molecule fluorescence lifetime imaging using a wide-field photon-counting detector. Proc SPIE Int Soc Opt Eng. (2009) 7185:1–10. doi: 10.1117/12.809496 PMC3103255 21625298

[47] Becker W Bergmann A In: Hickl B, editor. New SPCImage version combines time-domain analysis with phasor plot. Becker and Hickl GmbH. (2018).

[48] Malacrida L Ranjit S Jameson DM Gratton E. The phasor plot: a universal circle to advance fluorescence lifetime analysis and interpretation. Annu Rev Biophys. (2021) 50:575–93. doi: 10.1146/annurev-biophys-062920-063631 33957055

[49] Sauer L Schweitzer D Ramm L Augsten R Hammer M Peters S. Impact of macular pigment on fundus autofluorescence lifetimes. Invest Ophthalmol Vis Sci. (2015) 56:4668–79. doi: 10.1167/iovs.14-15335, PMID: 26207302

[50] Bernstein P Dysli C Fischer J Hammer M Katayama Y Sauer L. Fluorescence lifetime imaging ophthalmoscopy (FLIO) In: Bille JF, editor. High resolution imaging in microscopy and ophthalmology: new frontiers in biomedical optics. Cham: Springer. (2019). 213–35. 32091850

[51] Sauer L Andersen KM Li B Gensure RH Hammer M Bernstein PS. Fluorescence lifetime imaging ophthalmoscopy (FLIO) of macular pigment. Invest Ophthalmol Vis Sci. (2018) 59:3094–103. doi: 10.1167/iovs.18-23886, PMID: 30025128 PMC6009392

[52] Greenberg JP Duncker T Woods RL Smith RT Sparrow JR Delori FC. Quantitative fundus autofluorescence in healthy eyes. Invest Ophthalmol Vis Sci. (2013) 54:5684–93. doi: 10.1167/iovs.13-12445, PMID: 23860757 PMC3759220

[53] Dysli C Quellec G Abegg M Menke MN Wolf-Schnurrbusch U Kowal J. Quantitative analysis of fluorescence lifetime measurements of the macula using the fluorescence lifetime imaging ophthalmoscope in healthy subjects. Invest Ophthalmol Vis Sci. (2014) 55:2106–13. doi: 10.1167/iovs.13-13627, PMID: 24569585

[54] Hammer M Quick S Klemm M Schenke S Mata N Eitner A. In vivo and in vitro investigations of retinal fluorophores in age-related macular degeneration by fluorescence lifetime imaging In: Periasamy A Peter TC editors. Proc. SPIE BiOS (2009).

[55] Schweitzer D Gaillard ER Dillon J Mullins RF Russell S Hoffmann B. Time-resolved autofluorescence imaging of human donor retina tissue from donors with significant extramacular drusen. Invest Ophthalmol Vis Sci. (2012) 53:3376–86. doi: 10.1167/iovs.11-8970, PMID: 22511622 PMC3390004

[56] Wakita M Nishimura G Tamura M. Some characteristics of the fluorescence lifetime of reduced pyridine nucleotides in isolated mitochondria, isolated hepatocytes, and perfused rat liver in situ. J Biochem. (1995) 118:1151–60. doi: 10.1093/oxfordjournals.jbchem.a125001, PMID: 8720129

[57] Niesner R Peker B Schlüsche P Gericke KH. Noniterative biexponential fluorescence lifetime imaging in the investigation of cellular metabolism by means of NAD(P)H autofluorescence. ChemPhysChem. (2004) 5:1141–9. doi: 10.1002/cphc.200400066, PMID: 15446736

[58] Schneckenburger H Wagner M Weber P Strauss WSL Sailer R. Autofluorescence lifetime imaging of cultivated cells using a UV picosecond laser diode. J Fluoresc. (2004) 14:649–54. doi: 10.1023/B:JOFL.0000039351.09916.cc, PMID: 15617271

[59] Kierdaszuk B Malak H Gryczynski I Callis P Lakowicz JR. Fluorescence of reduced nicotinamides using one- and two-photon excitation. Biophys Chem. (1996) 62:1–13. doi: 10.1016/S0301-4622(96)02182-5, PMID: 8962467

[60] Andrews RM Griffiths PG Johnson MA Turnbull DM. Histochemical localisation of mitochondrial enzyme activity in human optic nerve and retina. Br J Ophthalmol. (1999) 83:231–5. doi: 10.1136/bjo.83.2.231, PMID: 10396204 PMC1722931

[61] Nakashima N Yoshihara K Tanaka F Yagi K. Picosecond fluorescence lifetime of the coenzyme of D-amino acid oxidase. J Biol Chem. (1980) 255:5261–3. doi: 10.1016/S0021-9258(19)70779-0, PMID: 6102996

[62] Skala MC Riching KM Gendron-Fitzpatrick A Eickhoff J Eliceiri KW White JG. In vivo multiphoton microscopy of NADH and FAD redox states, fluorescence lifetimes, and cellular morphology in precancerous epithelia. Proc Natl Acad Sci U S A. (2007) 104:19494–9. doi: 10.1073/pnas.0708425104, PMID: 18042710 PMC2148317

[63] Penjweini R Roarke B Alspaugh G Gevorgyan A Andreoni A Pasut A. Single cell-based fluorescence lifetime imaging of intracellular oxygenation and metabolism. Redox Biol. (2020) 34:101549. doi: 10.1016/j.redox.2020.101549, PMID: 32403080 PMC7217996

[64] Katz ML Drea CM Eldred GE Hess HH Robison WG Jr. Influence of early photoreceptor degeneration on lipofuscin in the retinal pigment epithelium. Exp Eye Res. (1986) 43:561–73. doi: 10.1016/S0014-4835(86)80023-9, PMID: 3792460

[65] Delori FC Goger DG Dorey CK. Age-related accumulation and spatial distribution of lipofuscin in RPE of normal subjects. Invest Ophthalmol Vis Sci. (2001) 42:1855–66. 11431454

[66] Bone RA Landrum JT Tarsis SL. Preliminary identification of the human macular pigment. Vis Res. (1985) 25:1531–5. doi: 10.1016/0042-6989(85)90123-3, PMID: 3832576

[67] Bone RA Landrum JT Hime GW Cains A Zamor J. Stereochemistry of the human macular carotenoids. Invest Ophthalmol Vis Sci. (1993) 34:2033–40. PMID: 8491553

[68] Snodderly DM Auran JD Delori FC. The macular pigment. II. Spatial distribution in primate retinas. Invest Ophthalmol Vis Sci. (1984) 25:674–85. PMID: 6724837

[69] Kijlstra A Tian Y Kelly ER Berendschot TTJM. Lutein: more than just a filter for blue light. Prog Retin Eye Res. (2012) 31:303–15. doi: 10.1016/j.preteyeres.2012.03.002, PMID: 22465791

[70] Sharifzadeh M Bernstein PS Gellermann W. Nonmydriatic fluorescence-based quantitative imaging of human macular pigment distributions. J Opt Soc Am A Opt Image Sci Vis. (2006) 23:2373–87. doi: 10.1364/JOSAA.23.002373, PMID: 16985523 PMC3079578

[71] Bhosale P Bernstein PS. Vertebrate and invertebrate carotenoid-binding proteins. Arch Biochem Biophys. (2007) 458:121–7. doi: 10.1016/j.abb.2006.10.005, PMID: 17188641 PMC1831825

[72] Loane E Nolan JM O'Donovan O Bhosale P Bernstein PS Beatty S. Transport and retinal capture of lutein and zeaxanthin with reference to age-related macular degeneration. Surv Ophthalmol. (2008) 53:68–81. doi: 10.1016/j.survophthal.2007.10.008, PMID: 18191658

[73] Bhosale P Larson AJ Frederick JM Southwick K Thulin CD Bernstein PS. Identification and characterization of a pi isoform of glutathione S-transferase (GSTP1) as a zeaxanthin-binding protein in the macula of the human eye. J Biol Chem. (2004) 279:49447–54. doi: 10.1074/jbc.M405334200, PMID: 15355982

[74] Bhosale P Bernstein PS. Synergistic effects of zeaxanthin and its binding protein in the prevention of lipid membrane oxidation. Biochim Biophys Acta. (2005) 1740:116–21. doi: 10.1016/j.bbadis.2005.02.002, PMID: 15949677

[75] Bhosale P Li B Sharifzadeh M Gellermann W Frederick JM Tsuchida K. Purification and partial characterization of a lutein-binding protein from human retina. Biochemistry. (2009) 48:4798–807. doi: 10.1021/bi9004478, PMID: 19402606

[76] Bernstein PS Balashov NA Tsong ED Rando RR. Retinal tubulin binds macular carotenoids. Invest Ophthalmol Vis Sci. (1997) 38:167–75. PMID: 9008641

[77] Bernstein PS Li B Vachali PP Gorusupudi A Shyam R Henriksen BS. Lutein, zeaxanthin, and meso-zeaxanthin: the basic and clinical science underlying carotenoid-based nutritional interventions against ocular disease. Prog Retin Eye Res. (2016) 50:34–66. doi: 10.1016/j.preteyeres.2015.10.003, PMID: 26541886 PMC4698241

[78] Woodall AA Britton G Jackson MJ. Carotenoids and protection of phospholipids in solution or in liposomes against oxidation by peroxyl radicals: relationship between carotenoid structure and protective ability. Biochim Biophys Acta. (1997) 1336:575–86. doi: 10.1016/S0304-4165(97)00007-X, PMID: 9367186

[79] Ham WT Jr Ruffolo JJ Jr Mueller HA Clarke AM Moon ME. Histologic analysis of photochemical lesions produced in rhesus retina by short-wave-length light. Invest Ophthalmol Vis Sci. (1978) 17:1029–35. PMID: 100464

[80] Krinsky NI. Antioxidant functions of carotenoids. Free Radic Biol Med. (1989) 7:617–35. doi: 10.1016/0891-5849(89)90143-3 2695406

[81] Bone RA Landrum JT Friedes LM Gomez CM Kilburn MD Menendez E. Distribution of lutein and zeaxanthin stereoisomers in the human retina. Exp Eye Res. (1997) 64:211–8. doi: 10.1006/exer.1996.0210, PMID: 9176055

[82] Sauer L Peters S Schmidt J Schweitzer D Klemm M Ramm L. Monitoring macular pigment changes in macular holes using fluorescence lifetime imaging ophthalmoscopy. Acta Ophthalmol. (2017) 95:481–92. doi: 10.1111/aos.13269, PMID: 27775222

[83] Sauer L Gensure RH Andersen KM Kreilkamp L Hageman GS Hammer M. Patterns of fundus autofluorescence lifetimes in eyes of individuals with nonexudative age-related macular degeneration. Invest Ophthalmol Vis Sci. (2018) 59:AMD65. doi: 10.1167/iovs.17-23764, PMID: 30025104 PMC6009207

[84] Dysli C Fink R Wolf S Zinkernagel MS. Fluorescence lifetimes of Drusen in age-related macular degeneration. Invest Ophthalmol Vis Sci. (2017) 58:4856–62. doi: 10.1167/iovs.17-22184, PMID: 28973332

[85] Sauer L Komanski CB Vitale AS Hansen ED Bernstein PS. Fluorescence lifetime imaging ophthalmoscopy (FLIO) in eyes with pigment epithelial detachments due to age-related macular degeneration. Invest Ophthalmol Vis Sci. (2019) 60:3054–63. doi: 10.1167/iovs.19-26835, PMID: 31348823 PMC6660189

[86] Hammer M Königsdörffer E Liebermann C Framme C Schuch G Schweitzer D. Ocular fundus auto-fluorescence observations at different wavelengths in patients with age-related macular degeneration and diabetic retinopathy. Graefes Arch Clin Exp Ophthalmol. (2008) 246:105–14. doi: 10.1007/s00417-007-0639-9, PMID: 17653752

[87] Schmidt J Peters S Sauer L Schweitzer D Klemm M Augsten R. Fundus autofluorescence lifetimes are increased in non-proliferative diabetic retinopathy. Acta Ophthalmol. (2017) 95:33–40. doi: 10.1111/aos.13174, PMID: 27519815

[88] Schweitzer D Deutsch L Klemm M Jentsch S Hammer M Peters S. Fluorescence lifetime imaging ophthalmoscopy in type 2 diabetic patients who have no signs of diabetic retinopathy. J Biomed Opt. (2015) 20:61106. doi: 10.1117/1.JBO.20.6.061106, PMID: 25769278

[89] Schweitzer D Quick S Klemm M Hammer M Jentsch S Dawczynski J. Time-resolved autofluorescence in retinal vascular occlusions. Ophthalmologe. (2010) 107:1145–52. doi: 10.1007/s00347-010-2195-7, PMID: 20552361

[90] Hammer M Sauer L Klemm M Peters S Schultz R Haueisen J. Fundus autofluorescence beyond lipofuscin: lesson learned from ex vivo fluorescence lifetime imaging in porcine eyes. Biomed Opt Express. (2018) 9:3078–91. doi: 10.1364/BOE.9.003078, PMID: 29984084 PMC6033583

[91] Dysli C Berger L Wolf S Zinkernagel MS. Fundus autofluorescence lifetimes and central serous chorioretinopathy. Retina. (2017) 37:2151–61. doi: 10.1097/IAE.0000000000001452, PMID: 28099314 PMC5690302

[92] Vitale AS Sauer L Modersitzki NK Bernstein PS. Fluorescence lifetime imaging ophthalmoscopy (FLIO) in patients with choroideremia. Transl Vis Sci Technol. (2020) 9:33–3. doi: 10.1167/tvst.9.10.33, PMID: 33062396 PMC7533737

[93] Dysli C Schuerch K Escher P Wolf S Zinkernagel MS. Fundus autofluorescence lifetime patterns in retinitis Pigmentosa. Invest Ophthalmol Vis Sci. (2018) 59:1769–78. doi: 10.1167/iovs.17-23336, PMID: 29610860

[94] Andersen KM Sauer L Gensure RH Hammer M Bernstein PS. Characterization of retinitis Pigmentosa using fluorescence lifetime imaging ophthalmoscopy (FLIO). Transl Vis Sci Technol. (2018) 7:20–18. doi: 10.1167/tvst.7.3.20, PMID: 29946494 PMC6016507

[95] Jaggi D Solberg Y Dysli C Ebneter A Wolf S Zinkernagel MS. Fluorescence lifetime imaging ophthalmoscopy: findings after surgical reattachment of macula-off Rhegmatogenous retinal detachment. Retina. (2020) 40:1929–37. doi: 10.1097/IAE.0000000000002718, PMID: 31860523 PMC7505146

[96] Sauer L Calvo CM Vitale AS Henrie N Milliken CM Bernstein PS. Imaging of Hydroxychloroquine toxicity with fluorescence lifetime imaging ophthalmoscopy. Ophthalmol Retina. (2019) 3:814–25. doi: 10.1016/j.oret.2019.04.025, PMID: 31345727

[97] Solberg Y Dysli C Möller B Wolf S Zinkernagel MS. Fluorescence lifetimes in patients with Hydroxychloroquine retinopathy. Invest Ophthalmol Vis Sci. (2019) 60:2165–72. doi: 10.1167/iovs.18-26079, PMID: 31108547

[98] Ermakov IV McClane RW Gellermann W Bernstein PS. Resonant Raman detection of macular pigment levels in the living human retina. Opt Lett. (2001) 26:202–4. doi: 10.1364/OL.26.000202, PMID: 18033547

[99] Charbel Issa P Gillies MC Chew EY Bird AC Heeren TFC Peto T. Macular telangiectasia type 2. Prog Retin Eye Res. (2013) 34:49–77. doi: 10.1016/j.preteyeres.2012.11.002, PMID: 23219692 PMC3638089

[100] Sauer L Gensure RH Hammer M Bernstein PS. Fluorescence lifetime imaging ophthalmoscopy: a novel way to assess macular telangiectasia type 2. Ophthalmol Retina. (2018) 2:587–98. doi: 10.1016/j.oret.2017.10.008, PMID: 30116796 PMC6089530

[101] Sauer L Vitale AS Modersitzki NK Bernstein PS. Longitudinal fluorescence lifetime imaging ophthalmoscopy analysis in patients with macular telangiectasia type 2 (MacTel). Retina. (2021) 41:1416–27. doi: 10.1097/IAE.0000000000003055, PMID: 34137386

[102] Sauer L Vitale AS Andersen KM Hart B Bernstein PS. Fluorescence lifetime imaging ophthalmoscopy (Flio) patterns in clinically unaffected children of macular telangiectasia type 2 (Mactel) patients. Retina. (2020) 40:695–704. doi: 10.1097/IAE.0000000000002646, PMID: 31517727 PMC7062574

[103] Solberg Y Dysli C Wolf S Zinkernagel MS. Fluorescence lifetime patterns in macular telangiectasia type 2. Retina. (2020) 40:99–108. doi: 10.1097/IAE.0000000000002411, PMID: 30664123 PMC6924947

[104] Goerdt L Sauer L Vitale AS Modersitzki NK Fleckenstein M Bernstein PS. Comparing fluorescence lifetime imaging ophthalmoscopy in atrophic areas of retinal diseases. Transl Vis Sci Technol. (2021) 10:11. doi: 10.1167/tvst.10.7.11, PMID: 34110387 PMC8196421

[105] Sauer L Vitale A Bernstein PS. The sensitivity and specificity of FLIO (fluorescence lifetime imaging ophthalmoscopy) when imaging patients with MacTel. Invest Ophthalmol Vis Sci. (2022) 63:42–2.35089328

[106] Michaelides M Hunt DM Moore AT. The genetics of inherited macular dystrophies. J Med Genet. (2003) 40:641–50. doi: 10.1136/jmg.40.9.641, PMID: 12960208 PMC1735576

[107] Stone EM Andorf JL Whitmore SS DeLuca AP Giacalone JC Streb LM. Clinically focused molecular investigation of 1000 consecutive families with inherited retinal disease. Ophthalmology. (2017) 124:1314–31. doi: 10.1016/j.ophtha.2017.04.008, PMID: 28559085 PMC5565704

[108] Allikmets R Singh N Sun H Shroyer NF Hutchinson A Chidambaram A. A photoreceptor cell-specific ATP-binding transporter gene (ABCR) is mutated in recessive Stargardt macular dystrophy. Nat Genet. (1997) 15:236–46. doi: 10.1038/ng0397-236, PMID: 9054934

[109] Boyer NP Higbee D Currin MB Blakeley LR Chen C Ablonczy Z. Lipofuscin and N-retinylidene-N-retinylethanolamine (A2E) accumulate in retinal pigment epithelium in absence of light exposure: their origin is 11-cis-retinal. J Biol Chem. (2012) 287:22276–86. doi: 10.1074/jbc.M111.329235, PMID: 22570475 PMC3381188

[110] Zhang N Tsybovsky Y Kolesnikov AV Rozanowska M Swider M Schwartz SB. Protein misfolding and the pathogenesis of ABCA4-associated retinal degenerations. Hum Mol Genet. (2015) 24:3220–37. doi: 10.1093/hmg/ddv073, PMID: 25712131 PMC4424957

[111] Cideciyan AV Aleman TS Swider M Schwartz SB Steinberg JD Brucker AJ. Mutations in ABCA4 result in accumulation of lipofuscin before slowing of the retinoid cycle: a reappraisal of the human disease sequence. Hum Mol Genet. (2004) 13:525–34. doi: 10.1093/hmg/ddh048, PMID: 14709597

[112] Dysli C Wolf S Hatz K Zinkernagel MS. Fluorescence lifetime imaging in Stargardt disease: potential marker for disease progression. Invest Ophthalmol Vis Sci. (2016) 57:832–41. doi: 10.1167/iovs.15-18033, PMID: 26934141

[113] Solberg Y Dysli C Escher P Berger L Wolf S Zinkernagel MS. Retinal flecks in Stargardt disease reveal characteristic fluorescence lifetime transition over time. Retina. (2019) 39:879–88. doi: 10.1097/IAE.0000000000002519, PMID: 30985551 PMC6510322

[114] Bambo MP Garcia-Martin E Pinilla J Herrero R Satue M Otin S. Detection of retinal nerve fiber layer degeneration in patients with Alzheimer's disease using optical coherence tomography: searching new biomarkers. Acta Ophthalmol. (2014) 92:e581–2. doi: 10.1111/aos.12374, PMID: 24592935

[115] Javaid FZ Brenton J Guo L Cordeiro MF. Visual and ocular manifestations of Alzheimer’s disease and their use as biomarkers for diagnosis and progression. Front Neurol. (2016) 7:55. doi: 10.3389/fneur.2016.00055 27148157 PMC4836138

[116] Kayabasi AU Sergott RC. OCT and FAF in the early diagnosis of Alzheimer's disease. Neurobiol Aging. (2014) 35:723. doi: 10.1016/j.neurobiolaging.2013.10.066

[117] Wang L Mao X. Role of retinal amyloid-β in neurodegenerative diseases: overlapping mechanisms and emerging clinical applications. Int J Mol Sci. (2021) 22:2360. doi: 10.3390/ijms22052360 33653000 PMC7956232

[118] Jentsch S Schweitzer D Schmidtke KU Peters S Dawczynski J Bär KJ. Retinal fluorescence lifetime imaging ophthalmoscopy measures depend on the severity of Alzheimer's disease. Acta Ophthalmol. (2015) 93:e241–7. doi: 10.1111/aos.12609, PMID: 25482990

[119] Sadda SR Borrelli E Fan W Ebraheem A Marion KM Harrington M. A pilot study of fluorescence lifetime imaging ophthalmoscopy in preclinical Alzheimer's disease. Eye (Lond). (2019) 33:1271–9. doi: 10.1038/s41433-019-0406-2, PMID: 30923356 PMC7005755

[120] Shivok K Affel E Sergott RC. Retinal mitochondrial fluorescence-lifetime ophthalmoscopy in patients with Alzheimer’s disease In: Alzheimer’s association international conference 2024, vol. 20. Philadelphia, PA: Wiley and Alzheimer’s Association (2024).

[121] Suh A Ong J Kamran SA Waisberg E Paladugu P Zaman N. Retina Oculomics in neurodegenerative disease. Ann Biomed Eng. (2023) 51:2708–21. doi: 10.1007/s10439-023-03365-0 37855949

[122] Rekik A Mrabet S Nasri A Abida Y Gharbi A Gargouri A. Eye movement study in essential tremor patients and its clinical correlates. J Neural Transm (Vienna). (2023) 130:537–48. doi: 10.1007/s00702-023-02614-9, PMID: 36894713

[123] Poewe W Seppi K Tanner CM Halliday GM Brundin P Volkmann J. Parkinson disease. Nat Rev Dis Primers. (2017) 3:17013. doi: 10.1038/nrdp.2017.13 28332488

[124] Ramirez AI de Hoz R Salobrar-Garcia E Salazar JJ Rojas B Ajoy D. The role of microglia in retinal neurodegeneration: Alzheimer's disease, Parkinson, and Glaucoma. Front Aging Neurosci. (2017) 9:214. doi: 10.3389/fnagi.2017.00214, PMID: 28729832 PMC5498525

[125] Li JL Lin TY Chen PL Guo TN Huang SY Chen CH. Mitochondrial function and Parkinson's disease: from the perspective of the Electron transport chain. Front Mol Neurosci. (2021) 14:797833. doi: 10.3389/fnmol.2021.797833, PMID: 34955747 PMC8695848

[126] Park JS Davis RL Sue CM. Mitochondrial dysfunction in Parkinson's disease: new mechanistic insights and therapeutic perspectives. Curr Neurol Neurosci Rep. (2018) 18:21. doi: 10.1007/s11910-018-0829-3, PMID: 29616350 PMC5882770

[127] Huang J Li Y Xiao J Zhang Q Xu G Wu G. Combination of multifocal Electroretinogram and spectral-domain OCT can increase diagnostic efficacy of Parkinson's disease. Parkinsons Dis. (2018) 2018:4163239. doi: 10.1155/2018/4163239 29755728 PMC5883930

[128] Unlu M Gulmez Sevim D Gultekin M Karaca C. Correlations among multifocal electroretinography and optical coherence tomography findings in patients with Parkinson's disease. Neurol Sci. (2018) 39:533–41. doi: 10.1007/s10072-018-3244-2, PMID: 29349656

[129] Shivok K Affel M Alizadeh T-W Liang D Kremens R Sergott R. Fluorescence-lifetime ophthalmoscopy findings in Parkinson’s disease patients with visual disturbances [abstract]. Mov Disord. (2023) 38:1.

[130] Miura Y Rauenbusch K Prasuhn J Brüggemann N Grisanti S Sonntag SR. Comparison of retinal fluorescence lifetime between patients with Parkinson’s disease and healthy subjects. Invest Ophthalmol Vis Sci. (2024) 65:1382–2.

[131] Shivok K Affel M Alizadeh T-W Liang D Kremens R Sergott R. Fluorescence lifetime imaging ophthalmoscopy (FLIO), a novel retinal mitochondrial biomarker for Parkinson’s disease. Mov Disord. (2024) 39:1. doi: 10.1002/alz.09188038294046

[132] Spaide RF. Retinal vascular cystoid macular edema: review and new theory. Retina. (2016) 36:1823–42. doi: 10.1097/IAE.0000000000001158, PMID: 27328171

[133] Nagelhus EA Mathiisen TM Ottersen OP. Aquaporin-4 in the central nervous system: cellular and subcellular distribution and coexpression with KIR4.1. Neuroscience. (2004) 129:905–13. doi: 10.1016/j.neuroscience.2004.08.053, PMID: 15561407

[134] Newman EA. Inward-rectifying potassium channels in retinal glial (Muller) cells. J Neurosci. (1993) 13:3333–45. doi: 10.1523/JNEUROSCI.13-08-03333.1993, PMID: 8340811 PMC6576530

[135] Sotirchos ES Saidha S Byraiah G Mealy MA Ibrahim MA Sepah YJ. In vivo identification of morphologic retinal abnormalities in neuromyelitis optica. Neurology. (2013) 80:1406–14. doi: 10.1212/WNL.0b013e31828c2f7a, PMID: 23516321 PMC3662269

[136] Foolad F Khodagholi F Nabavi SM Javan M. Changes in mitochondrial function in patients with neuromyelitis optica; correlations with motor and cognitive disabilities. PLoS One. (2020) 15:e0230691. doi: 10.1371/journal.pone.0230691, PMID: 32214385 PMC7098571

[137] Morch MT Khorooshi R Marczynska J Dubik M Nielsen S Nieland JD. Mitochondria-a target for attenuation of astrocyte pathology. J Neuroimmunol. (2021) 358:577657. doi: 10.1016/j.jneuroim.2021.577657, PMID: 34315069

[138] Cappellani F Sergott R Pulido J Affel E Fernandez B. NMOSD retinal mitochondrial fluorescence correlates with ganglion cell layer loss consistent with retinal Vasculitis, in ECTRIMS 2024. Copenhagen, DK: Sage Journals. (2024).

[139] Thiemann N Sonntag SR Kreikenbohm M Böhmerle G Stagge J Grisanti S. Artificial intelligence in fluorescence lifetime imaging ophthalmoscopy (FLIO) data analysis-toward retinal metabolic diagnostics. Diagnostics. (2024) 14:431. doi: 10.3390/diagnostics14040431, PMID: 38396470 PMC10888399

[140] Bowles Johnson KE Tang JAH Kunala K Huynh KT Parkins K Yang Q. Fluorescence lifetime imaging of human retinal pigment epithelium in pentosan polysulfate toxicity using adaptive optics scanning light ophthalmoscopy. Invest Ophthalmol Vis Sci. (2024) 65:27. doi: 10.1167/iovs.65.4.27, PMID: 38630675 PMC11044828

[141] Kunala K Tang JAH Bowles Johnson KE Huynh KT Parkins K Kim HJ. Near infrared autofluorescence lifetime imaging of human retinal pigment epithelium using adaptive optics scanning light ophthalmoscopy. Invest Ophthalmol Vis Sci. (2024) 65:27. doi: 10.1167/iovs.65.5.27, PMID: 38758638 PMC11107951

